# Geminiviral-induced genome editing using miniature CRISPR/Cas12j (CasΦ) and Cas12f variants in plants

**DOI:** 10.1007/s00299-023-03092-9

**Published:** 2024-02-19

**Authors:** Zheng Gong, Dominic Andrew Previtera, Yijie Wang, José Ramón Botella

**Affiliations:** Plant Genetic Engineering Laboratory, School of Agriculture and Food Sustainability, The University of Queensland, St Lucia, 4072 Australia

The efficient delivery of CRISPR reagents into plants is a significant bottleneck hindering the application of genome editing technologies. Viral vectors are an attractive alternative to traditional transformation as they can provide higher recombinant gene expression levels, increased efficiency and greater applicability due to the wide host range of some viruses (Gong et al. 2021). RNA viral vectors also enable transgene-free and potentially, seed-heritable genome editing, eliminating the need to transform and remove transgenes by out-crossing **(**Gong et al. 2021; Sukegawa et al. 2023**)**. However, many established plant DNA and positive-strand RNA viral delivery systems are hindered by a small cargo capacity for foreign genes, making them unsuitable to carry the popular CRISPR/Cas9 and Cas12a endonucleases **(**Sukegawa et al. 2023**)**. Hence, most developments using plant viral vectors have been limited to the delivery of CRISPR gRNAs into Cas transgenic plants as a proof-of-concept **(**Gong et al. 2021**)**.

The recent discovery of miniature type V CRISPR/Cas12j (CasΦ) and Cas12f systems, which are less than half the size of Cas9, may allow the delivery of entire CRISPR cassettes using size-restricted viral vectors. Several CRISPR/Cas12j and Cas12f systems have been characterized and shown to provide efficient genome editing in human cells **(**Bigelyte et al. 2021; Kim et al. 2022; Pausch et al. 2020; Wu et al. 2021; Xu et al. 2021**)**. Importantly, CRISPR/Cas12j-2 and the SpCas12f1 ortholog were shown to function in plants using conventional binary vectors or as ribonucleoproteins **(**Bigelyte et al. 2021; Pausch et al. 2020; Sukegawa et al. 2023**).** Other CRISPR/Cas12f orthologs, namely Un1Cas12f and AsCas12f1 have yet to be tested in plants. To the best of our knowledge, none of the miniature CRISPR systems have been delivered and evaluated using a plant viral vector.

Deconstructed geminiviral DNA replicons (GVRs) offer a robust viral-based platform for efficient delivery of genome editing reagents and donor DNA for gene targeting **(**Baltes et al. 2014). Although geminiviruses have been involved in the horizontal transfer of plant genetic material, their superior cargo capacity and high levels of recombinant gene expression make geminiviral replicons a suitable vector for transient expression of CRISPR components in *Nicotiana benthamiana* leaves (Catoni et al. 2018**).** Thus, we reasoned that geminiviral replicons could be a useful system for the rapid assessment of novel CRISPR systems. Here, we adopted a Bean yellow dwarf virus (BeYDV)-based geminiviral replicon (GVR) system, which delivers the geminiviral components using a binary vector, to evaluate the efficiency of novel miniature CRISPR/Cas12j-2 and Cas12f orthologs, SpCas12f1, AsCas12f1, and CasMINIv3.1 (protein-engineered Un1Cas12f variant) for plant genome editing. We first modified the GVR-based vector for the expression of gRNAs under the control of the *Arabidopsis* U6-26 promoter (collectively referred to as GVR-gRNA). Notably, we used gRNA scaffolds originally reported for Cas12j-2 and SpCas12f1, whilst engineered and improved gRNA scaffolds were used for AsCas12f1 and CasMINIv3.1 (Bigelyte et al. 2021; Kim et al. 2022; Pausch et al. 2020; Wu et al. 2021). A second GVR-based vector was then produced for expression of the different Cas proteins by deleting the geminiviral C1/C2 gene to increase the cargo capacity. Different *Cas12j/f* were cloned downstream of the cauliflower mosaic virus 35S promoter (collectively referred to as GVR-Cas). The HiBiT tag was added to all *Cas12j/f* constructs to allow quantification of the Cas12j/f proteins. Our experimental approach consisted in co-infiltrating *Nicotiana benthamiana* leaves with the appropriate GVR-gRNA and GVR-Cas vectors, allowing expression of proteins/gRNA by the geminiviral vectors for 6 – 8 days. The infiltrated tissues were analyzed to determine the relative abundance of the Cas proteins using HiBiT assays and genome editing by PCR amplification and amplicon sequencing (Fig. 1a).

**Fig. 1:**
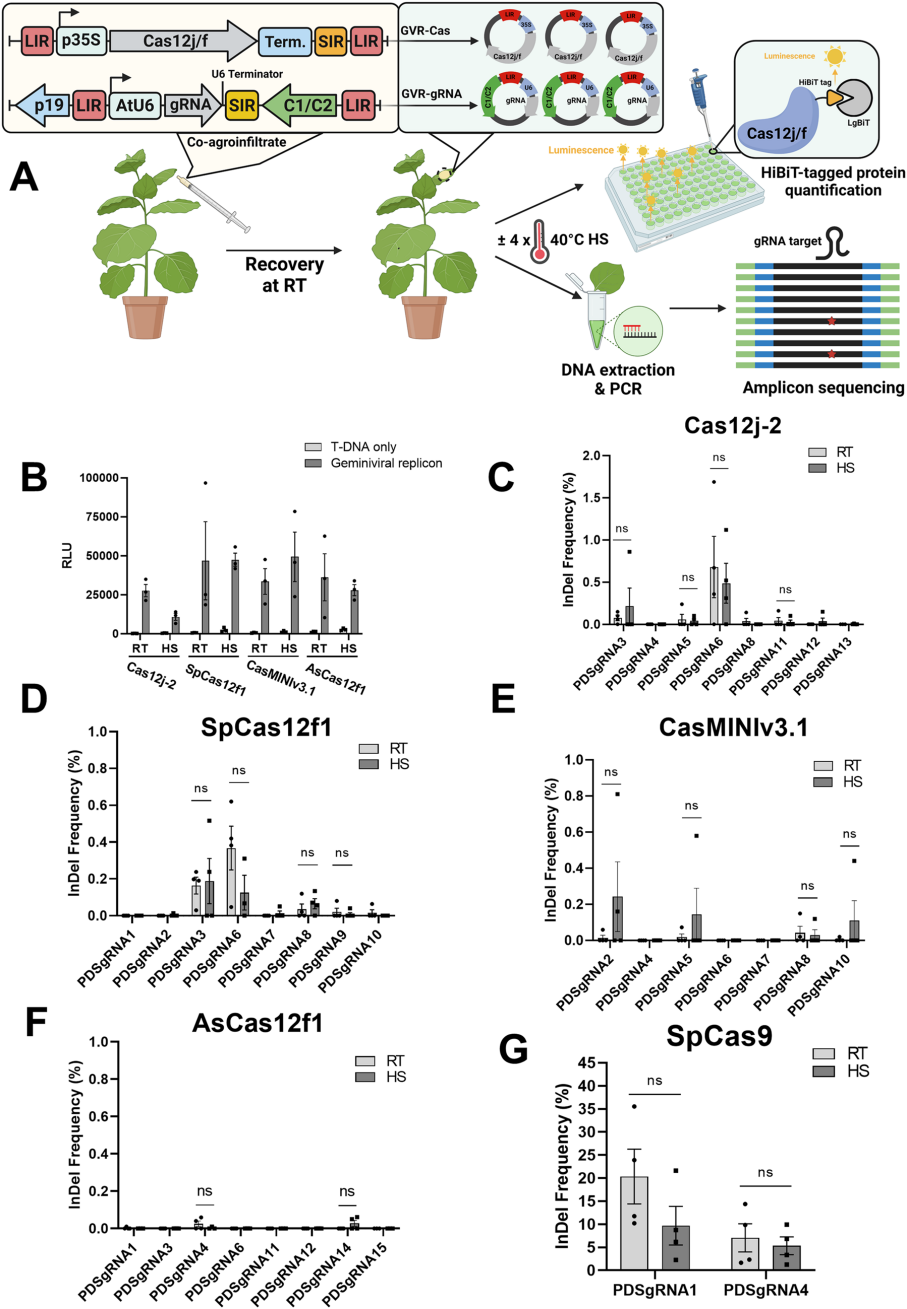
Evaluating miniature CRISPR/Cas12j and Cas12f orthologs for genome editing using a geminiviral replicon-based transient gene expression system, **a** Schematic representation of geminiviral replicon-based vectors and the workflow used to assess miniature CRISPR/Cas12j-2, SpCas12f1, AsCas12f1 and CasMINIv3.1 systems in plants (created with BioRender.com). The *Cas* endonuclease gene and the guide RNA (gRNA) were supplied in two different geminiviral-based constructs by co-agroinfiltration. Geminiviral replicons transiently express HiBiT-tagged Cas12j/f proteins. HiBiT-tagged protein levels were measured using a HiBiT bioluminescence assay on plants kept at room temperature (RT) or exposed to heat shock (HS). Alternatively, genomic DNA was extracted to detect CRISPR edits using amplicon sequencing. **b** HiBiT-tag bioluminescence assay of samples co-infiltrated with GVR-Cas and GVR-gRNA vectors (Geminiviral replicon) or GVR-Cas alone (T-DNA only). Agroinfiltrated plants were either kept at room temperature or subjected to HS (*N* = 3). Bars represent the mean relative luminescence units (RLU) ± SEM. **c** Insertion-deletion (InDel) frequency of transiently expressed Cas12j-2 using 8 gRNAs with plants kept at RT (*N* = 4 for all targets except *N* = 3 for gRNA13) or subjected to HS (*N* = 4 for all targets except *N* = 3 for gRNA8). **d** InDel frequency of transiently expressed SpCas12f1 using 8 gRNAs with plants kept at RT (*N* = 4) or subjected to HS (*N* = 4 for all gRNA targets except *N* = 5 for gRNA9 and *N* = 3 for gRNA6). **e** InDel frequency of transiently expressed CasMINIv3.1 using 7 gRNAs with plants kept at RT (*N* = 4) or subjected to HS (*N* = 4 for all gRNA targets except *N* = 3 for gRNA6). **f** InDel frequency of transiently expressed AsCas12f1 using 8 gRNAs with plants kept at RT (*N *= 4 for all gRNA targets except *N* = 3 for gRNA15) or subjected to HS (*N* = 4 for all gRNA targets except *N* = 3 for gRNA1, 4, 11, 15). **g** InDel frequency of transiently expressed SpCas9 using 2 sgRNAs with plants kept at RT (*N* = 4) or subjected to HS (N = 4). **c** to** g** Each bar represents the mean InDel frequency ± SEM. A parametric unpaired *t* test was used to test whether differences between InDel frequency of RT and HS samples were statistically significant. Only targets with at least two replicates across both treatments with detectable levels of CRISPR edits were analyzed. ns, no significant difference

The optimal temperature for in vitro dsDNA cleavage by Cas12f is in the range of ~ 40 °C to ~ 55 °C and providing high-temperature treatments was proven essential for SpCas12f1-mediated genome editing in maize **(**Bigelyte et al. 2021; Wu et al. 2021**)**, suggesting that short heat shocks may be necessary for efficient CRISPR/Cas12f function in plants. To test if heat shock exposure has a deleterious effect on the GVR expression system, we exposed co-infiltrated plants to a 4-h/40 °C heat shock (HS) for four consecutive days, while plants not subjected to HS were used as control. As a secondary control, we infiltrated leaves with only one of the two geminiviral vectors (GVR-Cas), which cannot form circularized geminiviral particles due to the absence of the C1/C2 gene but will drive expression of the different Cas proteins at the levels provided by regular binary vectors. As expected, protein levels produced by the geminiviral system (co-infiltrated tissues), were higher than those produced by the T-DNA controls (Fig. 1b). Most importantly, our results also show that the heat shock conditions used in our experimental design did not significantly affect the levels of Cas protein produced by the geminiviral system, compared to the no-HS treatment (Fig. 1b).

We cloned 8 different guides for Cas12j-2, SpCas12f1, AsCas12f1 and CasMINIv3.1 in the GVR-gRNA vectors targeting the *PHYTOENE DESATURASE* (*PDS*) gene in *N. benthamiana*. For comparative purposes, we also evaluated the editing efficiency of a GVR-SpCas9 construct using two known functional guide sequences targeting the *PDS* gene. *N. benthamiana* leaves were co-infiltrated with the respective GVR-Cas and GVR-gRNA vectors and either incubated at RT or subjected to the HS treatment described above. No bleaching phenotype, denoting complete knockout of the *PDS* alleles, was observed across samples. Targeted amplicon sequencing was used to detect CRISPR edits in the infiltrated tissue which yielded between 2,700 and 44,000 reads for each sample at the target loci (data not shown). We defined and analyzed the sequencing output using a stringent criterion (as described in Supplementary Methods) to eliminate noise from the sequencing results and account only for true edits. Compared to Cas9, all the studied Cas12 enzymes displayed very low genome editing efficiency in our hands (Fig. 1c to g). Cas12j-2 was the most efficient overall with gRNA6 producing ~ 0.7% and ~ 0.5% for the RT and HS treatments, respectively, which is comparable to the efficiency reported in *Arabidopsis* protoplasts (Pausch et al. 2020). 7 out of the 8 Cas12j-2 targets were functional, having at least one sample with detectable levels of InDels. SpCas12f1 was also functional at 6 out of 8 targets with the most efficient, gRNA6 mediating InDel frequencies of ~ 0.36% and ~ 0.12% for the RT and HS treatments, comparable with reports using transient expression of SpCas12f1 in maize (Bigelyte et al. 2021). We initially included an *N. benthamiana* codon optimized Un1Cas12f in our studies and failed to observe detectable levels of genome edits but decided to exclude it from our analysis due to the low protein expression levels detected by HiBiT-tag bioluminescence assays (data not shown). However, the protein engineered Un1Cas12f, CasMINIv3.1, was functional at 4 out of 7 targets but InDel frequencies were extremely variable. In addition, one of the guides used for CasMINIv3.1 did not meet our stringency requirements and was discarded from the final result analysis. Unfortunately, AsCas12f1, the smallest in size of all tested enzymes, failed to produce any detectable editing in 6 of the 8 tested targets and showed extremely low efficiency in the remaining two. CRISPR/SpCas9, on the other hand, mediated the production of InDels at frequencies ranging from 5.5% up to 20% across the two targets and temperature conditions. Surprisingly, heat shock treatments did not significantly improve genome editing efficiency for any of the Cas enzymes tested.

In-depth analysis of edits produced by Cas12j-2, CasMINIv3.1 and SpCas12f1 showed that deletions accounted for 97%, 99% and 100% of edits across all targets, respectively (Fig. S1). In contrast, genome editing outcomes of SpCas9 were balanced with 58.6% being deletions and 41.4% being insertions (Fig. S1). Most of the deletions produced by Cas12j-2, SpCas12f1 and CasMINIv3.1 were in the range of 5 bp to 15 bp with the most frequent being 9 bp, 10 bp and 9 bp, respectively (Fig. S2 and S3). These deletion patterns are typical of endonucleases that mediate staggered double-stranded DNA breaks. Meanwhile, most deletions produced by SpCas9 ranged from 1 to 7 bp (Figs. S2 and S3). We then analyzed the SpCas9, SpCas12f1 and Cas12j-2 deletion patterns at their most efficient target (Fig. S4). As expected, deletions produced by SpCas9 were concentrated 3 bp upstream from the 3’ end of the guide sequence. In contrast, deletions produced by SpCas12f1 and Cas12j-2 were spread across 5 to 6 bp, consistent with their larger deletion sizes. The highest deletion frequencies for SpCas12f1 were observed in the region from the 2nd bp upstream of the guide sequence to the 4th bp downstream while Cas12j-2 showed a similar pattern but shifted upstream by 2 bp. This information may assist in the selection of targets and parameters for assessing edits produced by Cas12j-2 and Cas12f in future plant genome editing experiments.

Bigelyte et al. (2021) reported that 18-nucleotide (nt) spacer/guide sequences were the most effective for SpCas12f1 in in vitro supercoiled plasmid cleavage assays. However, previous attempts at genome editing using SpCas12f1 in maize and rice were conducted using 20 nt guide sequences rather than 18 nt as used here (Bigelyte et al. 2021; Sukegawa et al. 2023). We, thus, selected the two most efficient SpCas12f1 gRNAs: gRNA3 and gRNA6 and extended them by 2 nt. The 20 nt and 18 nt guide sequences were used as guides and co-agroinfiltrated with GVR-SpCas12f1 on the same leaf for comparison. Analysis of the infiltrated tissues did not show significant differences in genome editing efficiency between the two spacer sequence lengths (Fig. S5).

The concentration of NaCl and MgCl_2_ greatly affects the performance of SpCas12f1 and AsCas12f1 in vitro while increased MgCl_2_ concentration in the tissue culture medium improved the number of genome-edited T_0_ transformants using CRISPR/Cas12j-2 (Cai et al. 2022). To study the effect of NaCl and MgCl_2_ on Cas12j-2, SpCas12f1, CasMINIv3.1 and AsCas12f1 editing efficiency, we selected the most efficient gRNA for each enzyme and evaluated editing efficiency in *N. benthamiana* leaves using two different concentrations of NaCl (100 mM and 200 mM) and MgCl_2_ (60 mM and 110 mM) in the infiltration buffer. The standard infiltration buffer, containing 10 mM MgCl_2_/0 mM NaCl, was used as a control and SpCas9 was included in the study for comparative purposes. Interestingly, no statistically significant differences were observed in Cas12j-2, SpCas12f1, CasMINIv3.1 and AsCas12f1 editing efficiency at any of the different NaCl and MgCl_2_ concentrations tested (Figs. S6 and S7). No significant differences were also observed for SpCas9 after treatment with both salts, suggesting that the inclusion of salts in the infiltration buffer might not be adequate for altering salt concentration in the cellular environment.

Overall, in this study, we assessed the capacity of AsCas12f1, SpCas12f1, CasMINIv3.1 and Cas12j-2 to mediate genome editing in plants. We utilized an effective workflow involving a geminiviral replicon-based platform to rapidly evaluate these newly discovered genome editing tools. Our results show that Cas12j-2, SpCas12f1 and CasMINIv3.1 are functional in plants and can be delivered using geminiviral vectors for genome editing, albeit at lower efficiencies than SpCas9. Further research is needed to increase the efficiency of the tested CRISPR systems or, alternatively, discovery and characterization of novel highly efficient miniature genome editors is essential for the use of RNA viral vectors in the development of transgene-free genome editing in plants. In addition, the availability of miniature Cas could be used to restrict the size of systems using Cas-fusion proteins such as base editors, prime editors and epigenome modifiers.

### Supplementary Information

Below is the link to the electronic supplementary material.

Supplementary file1 (PDF 990 KB)

## Declarations

### Data availability

Data supporting this study can be found in the Supplementary Information.
