# Supplementary material for: Geminiviral-induced genome editing using miniature CRISPR/Cas12j (CasΦ) and Cas12f variants in plants
Source: Plant Cell Rep. 2024 Feb 19;43(3):71. doi: 10.1007/s00299-023-03092-9 (PMC10876737; doi:10.1007/s00299-023-03092-9)
Supplement: Supplementary file 1 — Supplementary file1 (PDF 990 KB) [file 299_2023_3092_MOESM1_ESM.pdf]

# Geminiviral induced genome editing using miniature CRISPR/Cas12j (Casϕ) and Cas12f in plants.

**Zheng Gong**[1], Dominic Andrew Previtera[1], Yijie Wang[1], Jose Ramon Botella[1,*].

[1]Plant Genetic Engineering Laboratory, School of Agriculture and Food Sustainability, The University of Queensland, St Lucia, Australia, 4072.

***Correspondence:** Jose Ramon Botella (j.botella@uq.edu.au)

**ORCID:**
Zheng Gong (0000-0002-8554-7640)
Dominic Andrew Previtera (0009-0002-4859-0698)
Yijie Wang (0000-0002-1758-9558)
Jose Ramon Botella (0000-0002-4446-3432)

The document contains the following sections:
Supplementary results: Fig S1 – S7
Supplementary material and methods
Supplementary information

# Supplementary Results

This section contains the supplementary results, Fig S1 to S7 that support the findings of this study.

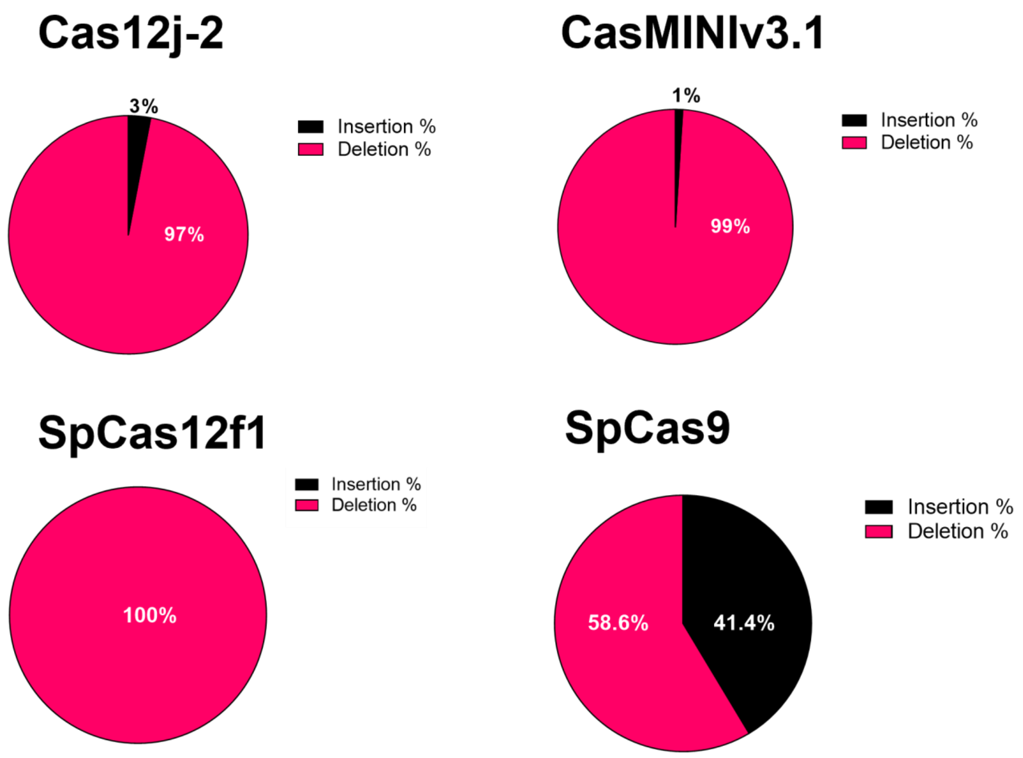


**Fig S1.** Percentage of insertions and deletions in the total mutations mediated by CRISPR/Cas12j- 2, SpCas12f1, CasMINIv3.1 and SpCas9 systems transiently expressed in *N. benthamiana* leaves using geminiviral replicons. The insertion/deletion percentage represents the frequency of insertion/deletion mutations identified across all targets divided by the sum of all InDels. The pie chart was produced for data across two targets for SpCas9, seven targets for CasMINIv3.1 and eight targets for Cas12j-2 and SpCas12f1.

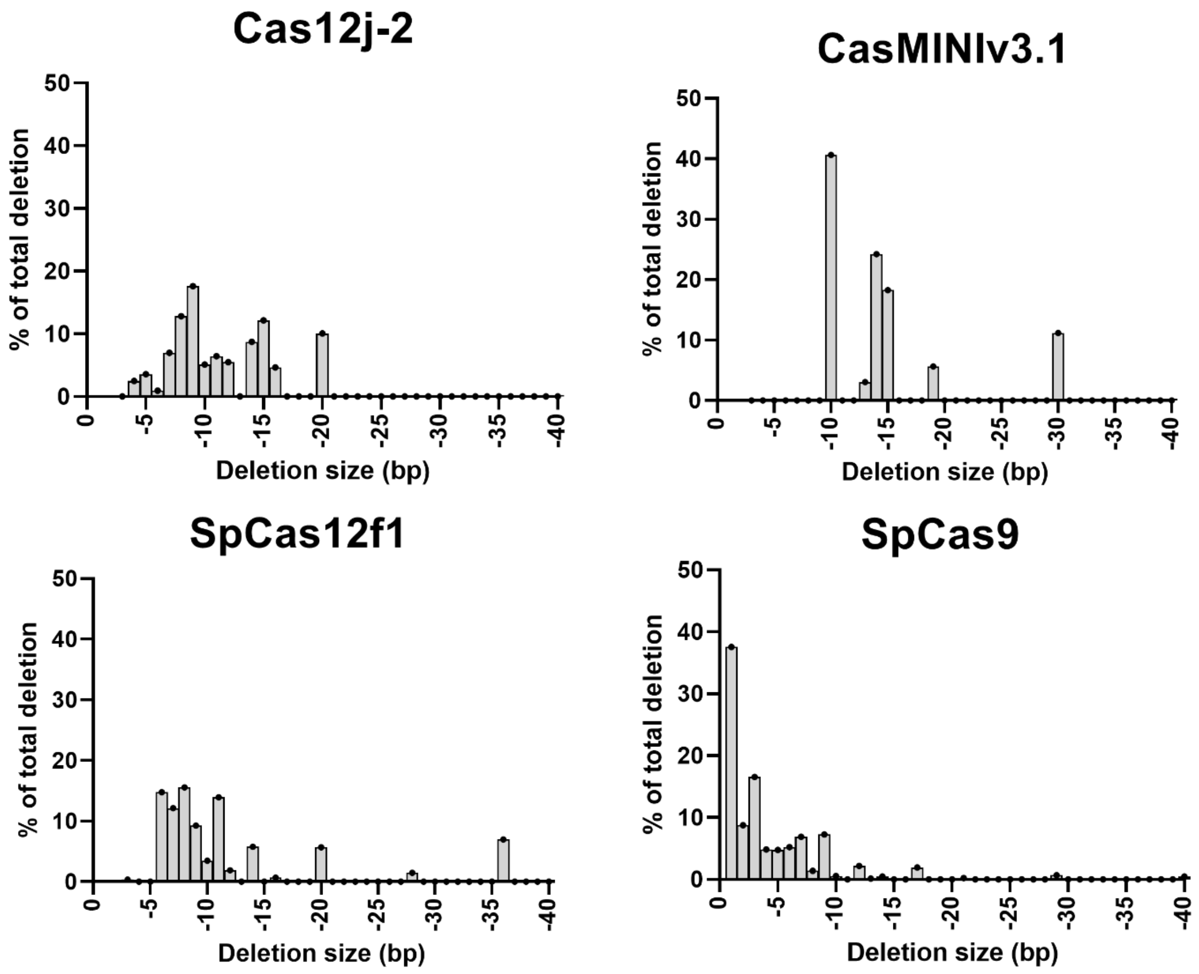


**Fig S2.** Deletion sizes of InDels produced by Cas12j-2, SpCas12f1, CasMINIv3.1 and SpCas9 across all targets used in this study. Deletion profiles were constructed for two targets for SpCas9, seven targets for CasMINIv3.1, and eight targets for Cas12j-2 and SpCas12f1. Values at each size were calculated as the percentage of edits with a particular deletion size divided by the total frequency of deletion mutations across all targets.

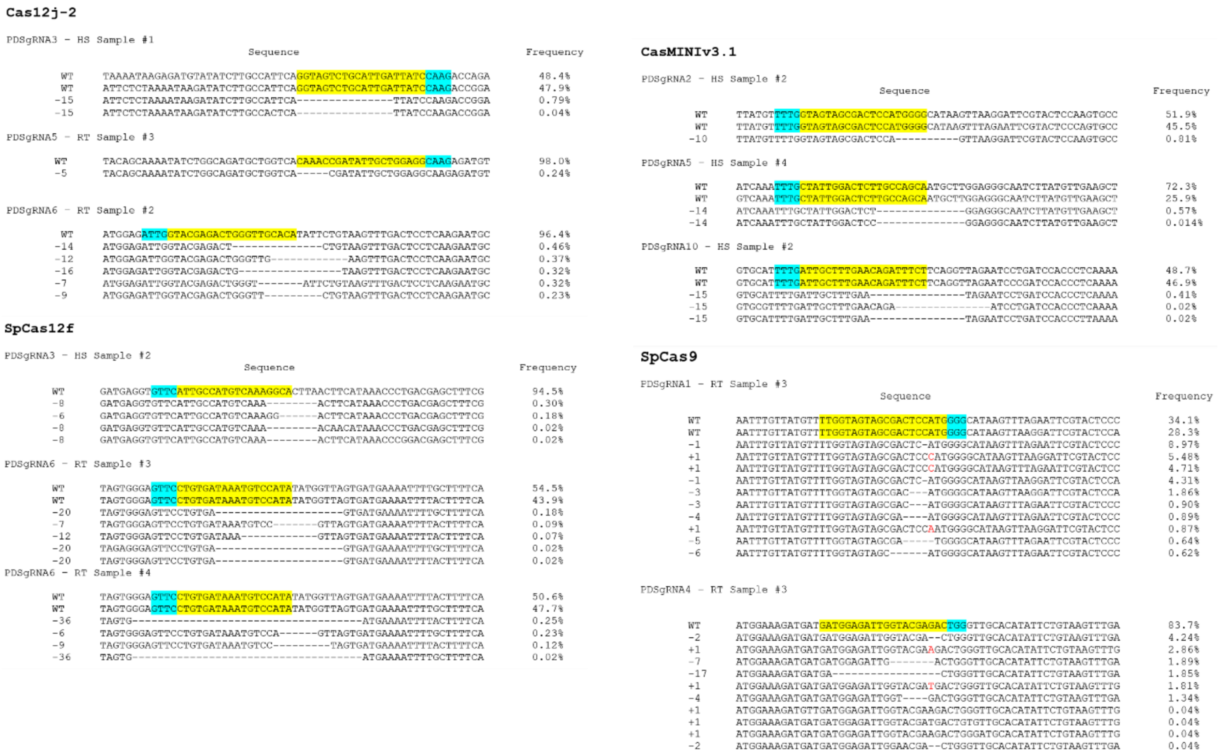


**Fig S3.** CRISPR/Cas12j-2, SpCas12f1, CasMINIv3.1 and SpCas9 mediated InDel profile across several targets. An individual sample was chosen for display. The spacer sequence is highlighted in yellow and the protospacer adjacent motif (PAM) is highlighted in blue. Deletions are marked as a dash, insertions are in red. WT, the wild-type allele/s. RT, room temperature treated samples. HS, heat shock treated samples.

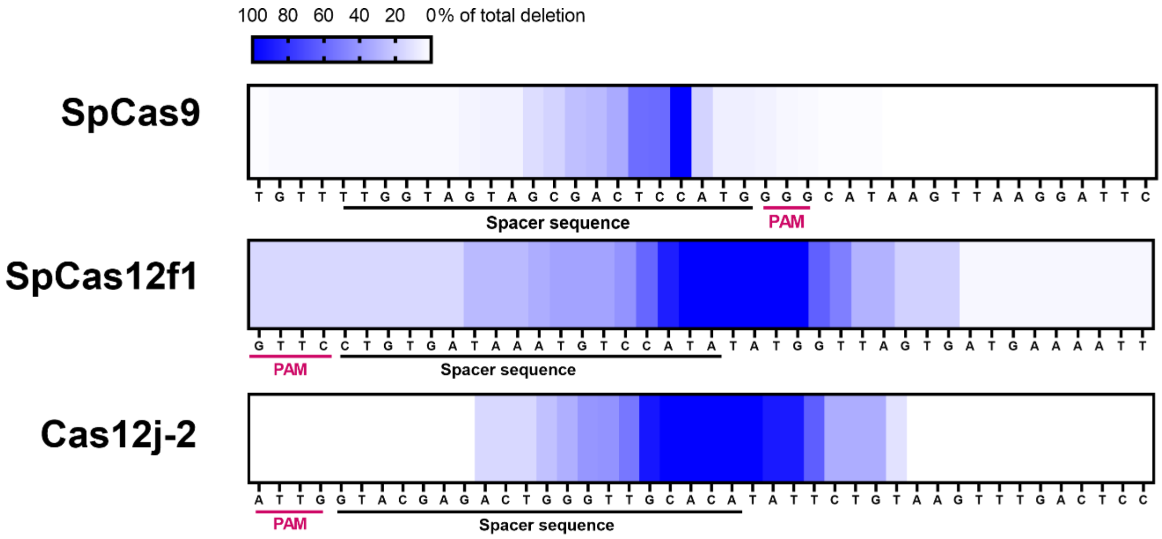


**Fig S4.** Comparison of Cas12j-2, SpCas12f1 and SpCas9 deletion patterns across the targeted region. The most efficient target was chosen for each CRISPR system. All samples (RT + HS) were combined in this analysis. 4 bp upstream and 20 bp downstream of the guide sequence were included in the plot. The spacer sequence is marked in black, and the PAM sequence is marked in pink. The percentage of deletion at a particular nucleotide is calculated as the frequency of deletions of the nucleotide divided by the total frequency of deletions at the target.

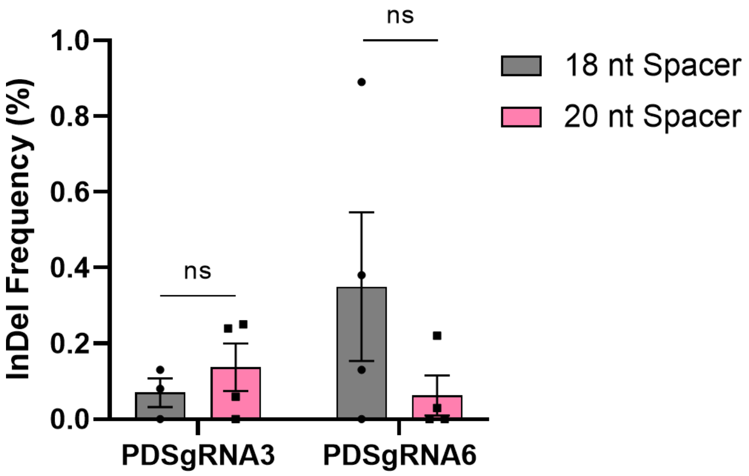


**Fig S5.** Effect of 18 nt and 20 nt spacer lengths on SpCas12f1 mediated genome editing. Two targets, gRNA3 (N = 3 for 18 nt spacer and N = 4 for 20 nt spacer) and gRNA6 (N = 4 for both spacer lengths) were evaluated. Each bar represents the mean InDel frequency ± SEM. A parametric unpaired t-test was used to test if differences between the frequency of InDels produced by SpCas12f1 with the two spacer lengths were statistically significant ($p < 0.05$). ns, no significant difference.

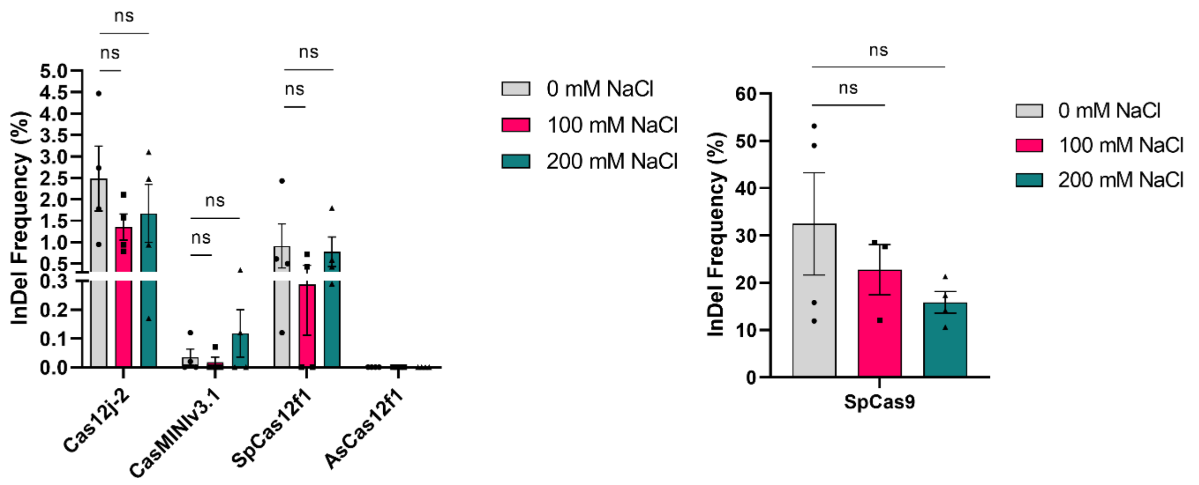


**Fig S6.** Effect of NaCl concentration on genome editing efficiency of miniature genome editors, Cas12j-2, CasMINIv3.1, SpCas12f1 and AsCas12f1 (left) and SpCas9 (right). Genome editing efficiency was measured using the default infiltration buffer (N = 4), infiltration buffer containing 100 mM NaCl (N = 4 except N = 3 for SpCas9), and infiltration buffer containing 200 mM NaCl (N = 4). Each bar represents the mean InDel frequency ± SEM. A parametric unpaired t-test was conducted between the default infiltration buffer and samples with increased NaCl concentration (100 mM or 200 mM) to determine if the differences were statistically significant ($p < 0.05$). ns, no significant difference.

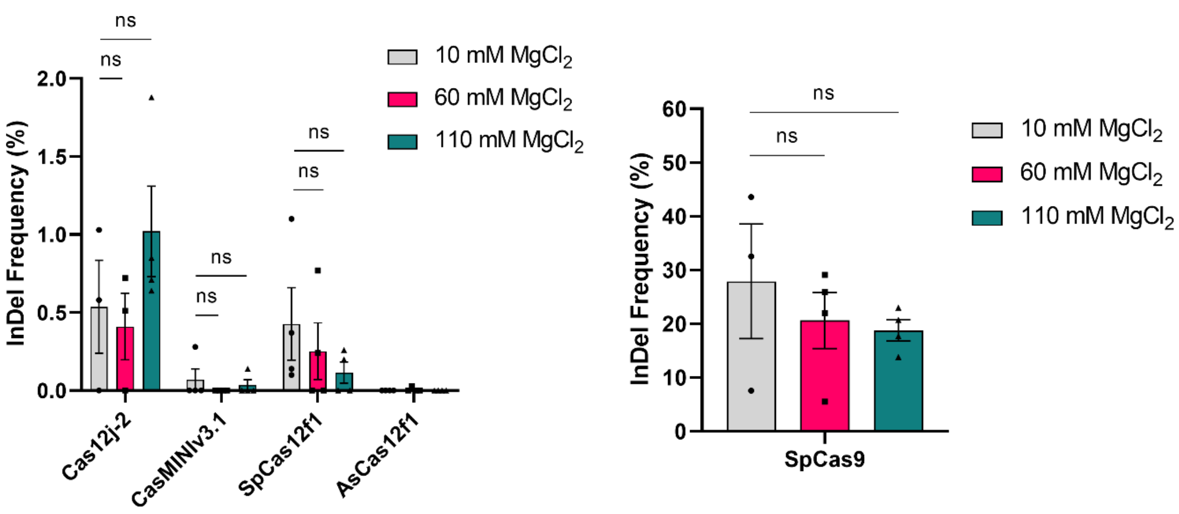


**Fig S7.** Effect of $MgCl_2$ concentration on genome editing efficiency of miniature genome editors, Cas12j-2, CasMINIv3.1, SpCas12f1 and AsCas12f1 (left) and SpCas9 (right). Genome editing efficiency was conducted between the default infiltration buffer containing 10 mM $MgCl_2$ (N = 3 for Cas12j-2 and SpCas9, N = 4 for all others), infiltration buffer containing 60 mM $MgCl_2$ (N = 3 for Cas12j-2, N = 4 for all others), and infiltration buffer containing 110 mM $MgCl_2$ (N = 4). Each bar represents the mean InDel frequency ± SEM. A parametric unpaired t-test was conducted between the default infiltration buffer and samples with increased $MgCl_2$ concentration (60 mM or 110 mM) to determine whether the differences were statistically significant ($p < 0.05$). ns, no significant difference.

## Supplementary Methods

This section contains the materials and methods used in this study.

*Vector construction.*

The pBYR2eFa GVR-based vector was originally constructed for recombinant protein expression (Diamos et al. 2016). Here, we modified it for Cas12j-2 and Cas12f gRNA expression (collectively referred to as GVR-gRNA). The *Arabidopsis* U6-26 small nucleolar RNA promoter was gene synthesized and cloned (Azenta Bioscience) into XhoI and ClaI digested pBYR2eFa, replacing the cauliflower mosaic virus (CaMV) 35S promoter, multiple cloning site and terminator to form pBYR2eFa-U6 (Table S1). The SpCas12f1, improved AsCas12f1 and Un1Cas12f gRNA scaffolds (referred to as SpCas12fgRNA, WugRNAv2 and KimgRNAv4.1, respectively) were gene synthesized and cloned (Azenta Bioscience) into pBYR2eFa-U6 using BsaI to form pBYR2eFa-U6-SpCas12fgRNA, pBYR2eFa-U6-WugRNAv2 and pBYR2eFa-U6-KimgRNAv4.1 (Table S1) (Bigelyte et al. 2021; Wu et al. 2021; Kim et al. 2022). The Cas12j-2 gRNA scaffold was synthesized as oligonucleotides (Sigma-Aldrich). The forward (5'-TGATTGCAACGATTGCCCCTCACGAGGGGACGGAGACCTTTGGTCTCC-3') and reverse (5'-AAAAGGAGACCAAAGGTCTCCGTCCCCTCGTGAGGGGCAATCGTTGCA-3') oligonucleotides were annealed in annealing buffer (10mM Tris at pH 7.5 - 8, 50 mM NaCl, 1 mM EDTA) and ligated into BsaI (New England Biolabs) digested pBYR2eFa-U6 to form pBYR2eFa-U6-Cas12jgRNA (Pausch et al. 2020). The SpCas9 sgRNA scaffold was PCR amplified using forward (5'-GAAGACCCTGATTGAGACCGGCCACGGGTCTCGGTTTTAGAGCTAGAAATAGCAAGTT–3') and reverse (5'–GGCGGAAGACCCAAAAGCACCGACTCGGTGCCA–3') primers with Q5 High Fidelity PCR Master Mix (New England Biolabs). The PCR amplicon was digested with BbsI (New England Biolabs) and ligated into BsaI digested pBYR2eFa-U6. Individual guide sequences (Table S2) targeting both alleles of the *PDS* gene (accession ID: Niben101Scf01283g02002; Niben101Scf14708g00023) were designed manually and synthesized as forward and reverse oligonucleotides (Sigma-Aldrich) (Bombarely et al. 2012). The oligonucleotides were mixed in annealing buffer, denatured at 95 degrees for 5 minutes and cooled to 25 degrees at a rate of 0.1 degrees per second. The annealed oligonucleotides were ligated into BsaI (New England Biolabs) digested pBYR2eFa-U6-SpCas12fgRNA, pBYR2eFa-U6-WugRNAv2, pBYR2eFa-U6-KimgRNAv4.1 or pBYR2eFa-U6-Cas12jgRNA accordingly. These vectors are referred to as GVR-gRNA for simplicity.

In addition, we also constructed pBYR2eFa- Cas12jPDSgRNA1-gRNA2, a dual Cas12j-2 gRNA cassette containing two guide sequences targeting the *PDS* gene multiplexed with glycine tRNAs and controlled under the 35S promoter (Xie et al. 2015). The dual Cas12j-2 gRNA cassette was subcloned into XhoI and XbaI digested pBYR2eFa. This vector was not used for genome editing but for the formation of geminiviral replicons in the HiBiT-tag bioluminescence assaying of Cas12j-2.

The pIZZA-BYR GVR-based vector was constructed for the co-expression of the CRISPR/Cas endonuclease gene alongside GVR-gRNA. The geminiviral long intergenic region (LIR), CaMV 35S

promoter, tobacco extensin 3’ UTR terminator, geminiviral short intergenic region (SIR) and another LIR were PCR amplified from pBYR2eFa using Q5 High Fidelity PCR Master Mix (New England Biolabs) (Diamos et al. 2016). PCR amplicons were assembled into SacI and HindIII (New England Biolabs) digested pCambia1300 using the NEBuilder HiFi DNA Assembly Master Mix (New England Biolabs). The *SpCas12f1*, *AsCas12f1* and *CasMINIv3.1* protein genes were gene synthesized with an HiBiT fusion (Promega) and cloned (Azenta Bioscience) into XbaI, SacI digested pIZZA-BYR. The *Cas12j-2* gene was PCR amplified from pPP537 (pPP537 was a gift from Jennifer Doudna (Addgene plasmid # 158803; http://n2t.net/addgene:158803; RRID: Addgene_158803)) using overhang primers that introduced the HiBiT sequence and assembled into pCambia1300 alongside the Cestrum yellow leaf curling virus (CmYLCV) promoter and *Arabidopsis* heat shock protein terminator (HSPt) using the NEBuilder HiFi DNA Assembly Master Mix (New England Biolabs). The *Cas12j-2* gene was PCR amplified from this vector and ligated into XbaI, SacI (New England Biolabs) digested pIZZA-BYR. The human codon optimized *SpCas9* protein gene was PCR amplified using forward primer (5’–ACAAACAACATTACAATTACTATTTACAATATGGCCCCAAAGAAGAAGCG–3’) and reverse primer (5’–ACCTTCAACTTTGTGATGTCACTTCGAGCTGAACGCATGCGGCCG–3’) and assembled into XbaI, SacI (New England Biolabs) digested pIZZA-BYR using the NEBuilder HiFi DNA Assembly Master Mix (New England Biolabs). All vectors were electroporated into electrocompetent *E. coli* cells and miniprepped through an in-house alkaline lysis miniprep method. All cloned plasmids were confirmed with colony PCR and Sanger sequencing (Australian Genome Research Facility).

*Plant materials*

LAB wildtype (WT) *Nicotiana benthamiana* plants were gifted by Prof. Peter Waterhouse. LAB WT seeds were directly sowed on UQ23 soil with 6 g/L Osmocote (UQ Plant Growth CRIP), germinated and maintained at 26 degrees with a 12-hour/12-hour day/night cycle. To ensure that plants withstood heat shocks, 6-week-old plants were used for all downstream experiments.

*Agroinfiltration of geminiviral replicon-based vectors for transient expression*

*Agrobacterium tumefaciens* GV3101 strain were electroporated with pIZZA-BYR-SpCas9, pIZZA-BYR-Cas12j-2, pIZZA-BYR-SpCas12f, pIZZA-BYR-AsCas12f and pIZZA-BYR-CasMINIv3.1 as well as the GVR-gRNA vectors with guide sequences cloned. Successfully transformed GV3101 *Agrobacterium* colonies were used to inoculate Lysogeny broth containing 100 mg/L Rifampicin and 50 mg/L Kanamycin. The GV3101 cultures were incubated at 26 degrees overnight, shaking at 250 RPM. Liquid cultures were centrifuged at 4000 RPM for 10 minutes, washed with infiltration buffer (10 mM $MgCl_2$, 10 mM MES pH = 5.6, 150 µM acetosyringone) and then pelleted at 4000 RPM for 10 minutes. *Agrobacterium* cultures were resuspended again in fresh infiltration buffer and were incubated at room temperature for 2 to 3 hours, in the dark. *Agrobacterium* transformed with GVR vectors containing the *SpCas9*, *Cas12j-2* or *Cas12f* genes were mixed with the

corresponding GVR-gRNA at an $OD_{600}$ of 0.1 for each GVR vector unless stated otherwise. The mixture was agroinfiltrated into the abaxial side of fully expanded leaves of six-week-old LAB WT *N. benthamiana* plants using a needleless syringe.

*Detection of protein expression with HiBiT tag bioluminescence assay*

To avoid tissues that become too necrotic to quantify protein expression, HiBiT bioluminescence assay was conducted at 6 days post agroinfiltration. LAB WT plants were agroinfiltrated with GVR-Cas12j/f vectors: pIZZA-BYR-SpCas12f, pIZZA-BYR-AsCas12f, pIZZA-BYR-CasMINIv3.1 or pIZZA-BYR-Cas12j-2. On the same leaf, the GVR-Cas12j/f was co-agroinfiltrated with the corresponding GVR-gRNA vectors: pBYR2eFa-U6-SpCas12fPDSgRNA3, pBYR2eFa-U6-WuPDSgRNA1, pBYR2eFa-U6-KimPDSgRNA2 or pBYR2eFa-Cas12jPDSgRNA1-gRNA2, respectively. Agroinfiltrated plants were recovered at room temperature for two days and then subjected to either four consecutive days of 4-hour, 40-degree heat shocks or remained at room temperature. Leaf discs were collected into 96 well microplates after the last heat shock with a short period for recovery (4 to 6 hours). Three leaf discs were collected for each agroinfiltrated leaf and HiBiT-tagged protein levels were quantified using the Nano-Glo HiBiT Lytic Detection System (Promega). A HiBiT luminescence master mix was prepared whereby the HiBiT lytic substrate and LgBiT protein were diluted together by 50 or 100 times, respectively, in room temperature HiBiT lytic buffer. 20 µl of the HiBiT master mix was directly pipetted onto the surface of leaf discs and incubated at room temperature for 10 minutes. Bioluminescence was measured using lens filter on the BMG FLUOstar Omega Microplate Reader for 30 cycles. The relative luminescence unit of the last cycle was recorded. RLU for the three leaf discs collected from one agroinfiltration event were averaged to obtain the final RLU.

*Genome editing with miniature CRISPR/Cas12j and Cas12f systems transiently expressed using geminiviral replicons.*

Plants co-agroinfiltrated with GVR-Cas12j/f or GVR-SpCas9 as control and its corresponding GVR-gRNA vector were recovered at room temperature for three days. Treated plants were then subjected to either four consecutive days of 4-hour, 40-degree heat shocks or remained at room temperature. After 24 hours of recovery, leaf discs were collected from agroinfiltrated regions of all plants. Genomic DNA was extracted according to Edwards et al. (1991) with the exception that after the removal of isopropanol, the DNA pellet was washed again with 70% ethanol. The pellet was resuspended in 40 µl of RNase-free water instead of 100 µl of TE buffer (Edwards et al. 1991). Regions around the gRNA target were PCR amplified using Q5 High Fidelity PCR Master Mix (New England Biolabs) with Nextera-tagged primers (Table S3). Two amplicons with different sequences were mixed at equal volumes to a total of 20 µl and sent for 150 bp paired-end Illumina MiSeq sequencing (Australian Genome Research Facility). The reads were mapped to the reference and analyzed for

CRISPR edits using CRISPResso2 with average read quality of Phred33 scale >20, single bp quality >10. Samples with <1000 reads mapped to the reference sequence were excluded from the analysis (Clement et al. 2019).

For the detection of Cas9-induced InDels, the default Cas9 setting was used. Substitutions were ignored to specifically identify InDels. Some InDels were detected in negative controls (leaves infiltrated with infiltration buffer) which may be due to contaminants, PCR amplification or sequencing error. Thus, the final InDel frequency was calculated as the sample's InDel frequency minus the frequency of the same InDel type in the control. For detection of miniature CRISPR/Cas12j or Cas12f-induced InDels, the center of quantification window was set as 0 nucleotides from the 3' end of guide sequences, the quantification window was set to 10 nucleotides and a window size of 30 nucleotides were graphed. Likewise, substitutions were intentionally ignored to only detect and quantify InDels. A more stringent criterion was used to filter out the noise and obtain true CRISPR-induced InDels. We manually eliminated any InDels ≤2 bp due to their presence in control samples, similar to what was previously used (Pausch et al. 2020). Additionally, InDels were also completely excluded if the InDel type was present in the negative control despite its size. The final InDel frequency was calculated based on these criteria. An unpaired parametric t-test was used to determine if differences in genome editing efficiency between RT and HS treatments are statistically significant ($p < 0.05$). gRNAs which had at least two replicates with detectable CRISPR InDels between both temperature treatments were analyzed.

The InDel patterns of each CRISPR/Cas system were manually extracted from the CRISPResso2 output files. Insertion and deletion ratios were calculated as a percentage of total InDels across all targets evaluated. It is to note that InDels of ≤2 bp in size were excluded from analysis for all CRISPR/Cas12j or Cas12f systems. All deletions obtained for a particular CRISPR/Cas system, across all of its targets, were categorized based on size and calculated as a percentage of total deletion. To analyze the distribution and pattern of deletions across the target, we selected the most efficient gRNA for SpCas12f1, Cas12j-2 and SpCas9. We combined samples from both RT and HS treatments and calculated the deletion frequency at each nucleotide as a percentage of total deletion obtained for the gRNA. We evaluated the 4 bp upstream, which contains the PAM of SpCas12f1 and Cas12j-2, up to 20 bp downstream of the target sequence.

*Comparison of genome editing efficiency between 18 and 20 nucleotide spacer sequences for the CRISPR/SpCas12f1 system.*

The two most efficient gRNAs, gRNA3 and gRNA6 were selected for testing. The spacer sequence of the two gRNAs was extended by 2 nt on the 3' end. The 20 nt spacer sequences were cloned into pBYR2eFa-U6-SpCas12fgRNA as above to produce pBYR2eFa-U6-SpCas12fPDSgRNA3-20nt and pBYR2eFa-U6-SpCas12fPDSgRNA6-20nt. The GVR-gRNA vectors containing the 18 nt and 20 nt spacer sequences were electroporated into electrocompetent GV3101 and prepared for

agroinfiltration. The pIZZA-BYR-SpCas12f was mixed with the GVR-gRNA containing the 18 nt or 20 nt spacer sequence and was agroinfiltrated side by side onto different regions of the same leaf. Genomic DNA was extracted from the agroinfiltrated leaves on the eighth day. Samples were prepared and sent for targeted amplicon sequencing as above (Australian Genome Research Facility). The output was analyzed using CRISPResso2 following previously described parameters and criteria. An unpaired parametric t-test was used to determine if differences between InDel frequencies generated by 18 nt and 20 nt spacer sequences are statistically significant ($p < 0.05$).

*Effect of NaCl and $MgCl_2$ salt concentration on CRISPR/Cas12j-2 and Cas12f genome editing efficiency.*

To investigate the effect of NaCl and $MgCl_2$ salt concentration on the efficiency of CRISPR/Cas12j and Cas12f-mediated genome editing in plants, we selected the most effective gRNA: gRNA6 for SpCas12f1, gRNA2 for CasMINIv3.1 and gRNA6 for Cas12j-2. For AsCas12f1, gRNA1 was selected as it has the same guide sequence as the CasMINIv3.1 gRNA2 and is similar to sgRNA1 for SpCas9. sgRNA1 of SpCas9 was used as a control. GV3101 *Agrobacterium* cultures were prepared for agroinfiltration as above. However, after mixing GVR-Cas with the corresponding GVR-gRNA vector in the infiltration buffer, NaCl solution was added to a concentration of either 0 mM, 100 mM, or 200 mM (the infiltration buffer also contains 10 mM $MgCl_2$) or $MgCl_2$ solution was added to a concentration of 10 mM (default concentration of infiltration buffer), 60 mM or 110 mM. The *Agrobacterium* mixtures containing three different concentrations of salt were agroinfiltrated into different regions of the same leaf of 6-week-old LAB WT plants. Treated plants were maintained at RT and leaf discs were collected 8 days post agroinfiltration. Genomic DNA was extracted for PCR amplification and Illumina-based amplicon sequencing (Australian Genome Research Facility). InDels were identified using CRISPResso2, following the same criteria as described above. An unpaired parametric t-test was used to determine whether differences between the frequency of InDels in samples infiltrated with increased salt concentrations and the default infiltration buffer (0 mM NaCl and 10 mM $MgCl_2$) are statistically significant ($p < 0.05$).

*Data analysis.*

All statistical analyses were conducted using GraphPad Prism version 9.3.1.

## Supplementary Information

This section contains Supplementary information that supports the findings of this study, including construct, primer, and guide sequences.

**Table S1. Vector name, description, and sequence.**

| Plasmid name | Description |
|---|---|
| pIZZA-BYR | Geminiviral replicon-based vector with the geminiviral C1/C2 genes deleted for *Cas12j/f* protein gene expression. |
| pIZZA-BYR-SpCas12f | Geminiviral replicon-based vector for transient expression of *SpCas12f1* protein gene. |
| pIZZA-BYR-AsCas12f | Geminiviral replicon-based vector for transient expression of *AsCas12f1* protein gene. |
| pIZZA-BYR-CasMINIv3.1 | Geminiviral replicon-based vector for transient expression of *CasMINIv3.1* protein gene. |
| pIZZA-BYR-Cas12j-2 | Geminiviral replicon-based vector for transient expression of *Cas12j-2 (Casϕ-2)* protein gene. |
| pIZZA-BYR-hSpCas9 | Geminiviral replicon-based vector for transient expression of *hSpCas9* protein gene. |
| pBYR2eFa-U6 | Geminiviral replicon-based vector containing an *Arabidopsis* U6-26 promoter with multiple cloning site (MCS). |
| pBYR2eFa-U6-SpCas12fgRNA | Geminiviral replicon-based vector containing an *Arabidopsis* U6-26 promoter and the SpCas12f1 gRNA scaffold with type IIS restriction enzyme for cloning guide sequences. |
| pBYR2eFa-U6-WugRNAv2 | Geminiviral replicon-based vector containing an *Arabidopsis* U6-26 promoter and the improved AsCas12f1 gRNA scaffold (WugRNAv2) with type IIS restriction enzyme for cloning guide sequences. |
| pBYR2eFa-U6-KimgRNAv4.1 | Geminiviral replicon-based vector containing an *Arabidopsis* U6-26 promoter and the improved Un1Cas12f gRNA scaffold (KimgRNAv4.1) with type IIS restriction enzyme for cloning guide sequences. This vector was used for the expression of CasMINIv3.1 gRNAs. |
| pBYR2eFa-U6-Cas12jgRNA | Geminiviral replicon-based vector containing an *Arabidopsis* U6-26 promoter and the processed 25 nt Cas12j-2 gRNA scaffold with type IIS restriction enzyme for cloning guide sequences. |
| pBYR2eFa-Cas12jPDSgRNA1-gRNA2 | Geminiviral replicon-based vector containing a CaMV 35S promoter with glycine tRNA for processing of two Cas12j-2 gRNAs targeting the *PDS* gene. This vector was used for assaying HiBiT-tag bioluminescence in combination with pIZZA-BYR-Cas12j-2. |
| pBYR2eFa-U6-sgRNArepeat | Geminiviral replicon-based vector containing an *Arabidopsis* U6-26 promoter and SpCas9 sgRNA scaffold with type IIS restriction enzyme for cloning guide sequences. |

## Sequence information

**pIZZA-BYR:** Geminiviral long intergenic region (LIR) is marked in yellow, geminiviral short intergenic region (SIR) is marked in grey, Cauliflower mosaic virus 35S promoter is marked in blue, tobacco mosaic virus 5' UTR is marked in green, tobacco extensin 3' UTR terminator is marked in pink. Restriction enzyme sites where the *Cas12f/j* protein genes were cloned in between are underlined. Nucleotides where the SpCas9 protein gene was assembled in between, are **bolded**.

ACGAATTCCTTCCTTTAGCAGCCCTTGCGCCCTGAGTGCTTGCGGCAGCGTGAAGCTGGCGCGCCGCTCTAGCAGAAGGCATGTTGTTGTGACTCCGAGGGGTTGCCTCA
AACTCTATCTTATAACCGGCGTGGAGGCATGGAGGCAAGGGCATTTTGGTAATTTAAGTAGTTAGTGGAAAATGACGTCATTTACTTAAAGACGAAGTCTTGCGACAAGG
GGGGCCCACGCCGAATTTTAATATTACCGGCGTGGCCCCACCTTATCGCGAGTGCTTTAGCACGAGCGGTCCAGATTTAAAGTAGAAAAGTTCCCGCCCACTAGGGTTAA
AGGTGTTCACACTATAAAAGCATATACGATGTGATGGTATTTGATGGAGCGTATATTGTATCAGGTATTTCCGTCGGATACGAATTATTCGTACGACCCTCCTGCAGGTCA
ACATGGTGGAGCACGACACACTTGTCTACTCCAAAAATATCAAAGATACAGTCTCAGAAGACCAAAGGGCAATTGAGACTTTTCAACAAAGGGTAATATCCGGAAACCT
CCTCGGATTCCATTGCCCAGCTATCTGTCACTTTATTGTGAAGATAGTGGAAAAGGAAGGTGGCTCCTACAAATGCCATCATTGCGATAAAGGAAAGGCCATCGTTGAAG
ATGCCTCTGCCGACAGTGGTCCCAAAGATGGACCCCCACCCACGAGGAGCATCGTGGAAAAAGAAGACGTTCCAACCACGTCTTCAAAGCAAGTGGATTGATGTGATAT
CTCCACTGACGTAAGGGATGACGCACAATCCCACTATCCTTCGCAAGACCCTTCCTCTATATAAGGAAGTTCATTTCATTTGGAGAGGACCTCGAGTATTTTTACAACAAT
TACCAACAACAACAAACAACAAACAACATTACAATTACTATTTACAA **T**CTAGACCCGG**GAGCTC**GAAGTGACATCACAAAGTTGAAGGTAATAAAGCCAAAT
TAATTAAGACATTTTCATAATGATGTCAAGAATGCAAAGCAAATTGCATAACTGCCTTTATGCAAAACATTAATATAATATAAATTATAAAGAACTGCGCTCTCTGCTTCT
TATTTTCTTAGCTTCATTTATTAGTCACTAGCTGTTCAGAATTTTCAGTATCTTTTGATATTACTAAGAACCTAATCACACAATGTATATTCTTATGCAGGAAAAGCAGAAT
GCTGAGCTAAAAGAAAGGCTTTTCCATTTTCGAGAGACAATGAGAAAAGAAGAAGAAGAAGAAGAAGAAGAAGAAGAAAAGAGTAAATAATAAAGCCCCACAG
GAGGCGAAGTTCTTGTAGCTCCATGTTATCTAAGTTATTGATATTGTTTGCCCTATATTTTATTTCTGTCATTGTGTATGTTTTGTTCAGTTTCGATCTCCTTGCAAAATGCA
GAGATTATGAGATGAATAAACTAAGTTATATTATTATACGTGTTAATATTCTCCTCCTCTCTCTAGCTAGCCTTTTGTTTTCTCTTTTTCTTATTTGATTTTCTTTAAATCAAT
CCATTTTAGGAGAGGGCCAGGGAGTGATCCAGCAAAACATGAAGATTAGAAGAAACTTCCCTCTTTTTTTTCCTGAAAACAATTTAACGTCGAGATTTATCTCTTTTTGTA
ATGGAATCATTTCTACAGTTATGACCCTAGGCGAGTGTACTTCAAGTCAGTTGGAAATCAATAAAATGATTATTTTATGAATATATTTCATTGTGCAAGTAGATAGAAATT
ACATATGTTACATAACACACGAAATAAACAAAAAAACACAATCCAAAACAAACACCCCAAACAAAATAACACTATATATATCCTCGTATGAGGAGAGGCACGTTCAGT
GACTCGACGATTCCCGAGCAAAAAAAGTCTCCCCGTCACACATATAGTGGGTGACGCAATTATCTTCAAAGTAATCCTTCTGTTGACTTGTCATTGATAACATCCAGTCTT
CGTCAGGATTCCAAAGAATTATAGAAGGGATCCTAGCAGAAGGCATGTTGTTGTGACTCCGAGGGGTTGCCTCAAACTCTATCTTATAACCGGCGTGGAGGCATGGAGG
CAAGGGCATTTTGGTAATTTAAGTAGTTAGTGGAAAATGACGTCATTTACTTAAAGACGAAGTCTTGCGACAAGGGGGGCCCACGCCGAATTTTAATATTACCGGCGTGG
CCCCACCTTATCGCGAGTGCTTTAGCACGAGCGGTCCAGATTTAAAGTAGAAAAGTTCCCGCCCACTAGGGTTAAAGGTGTTCACACTATAAAAGCATATACGATGTGAT
GGTATTTGATGGAGCGTATATTGTATCAGGTATTTCCGTCGGATACGAATTATTAAGCTTGGCACTGGCCGTCGTTTTACAACGTCGTGACTGGGAAAACCCTGGCGTTAC
CCAACTTAATCGCCTTGCAGCACATCCCCCTTTCGCCAGCTGGCGTAATAGCGAAGAGGCCCGCACCGATCGCCCTTCCCAACAGTTGCGCAGCCTGAATGGCGAATGCT
AGAGCAGCTTGAGCTTGGATCAGATTGTCGTTTCCCGCCTTCAGTTTAAACTATCAGTGTTTGACAGGATATATTGGCGGGTAAACCTAAGAGAAAAGAGCGTTTATTAG
AATAACGGATATTTAAAAGGGCGTGAAAAGGTTTATCCGTTCGTCCATTTGTATGTGCATGCCAACCACAGGGTTCCCCTCGGGATCAAAGTACTTTGATCCAACCCCTC
CGCTGCTATAGTGCAGTCGGCTTCTGACGTTCAGTGCAGCCGTCTTCTGAAAACGACATGTCGCACAAGTCCTAAGTTACGCGACAGGCTGCCGCCCTGCCCTTTTCCTGG
CGTTTTCTTGTCGCGTGTTTTAGTCGCATAAAGTAGAATACTTGCGACTAGAACCGGAGACATTACGCCATGAACAAGAGCGCCGCCGCTGGCCTGCTGGGCTATGCCCG
CGTCAGCACCGACGACCAGGACTTGACCAACCAACGGGCCGAACTGCACGCGGCCGGCTGCACCAAGCTGTTTTCCGAGAAGATCACCGGCACCAGGCGCGACCGCCCG
GAGCTGGCCAGGATGCTTGACCACCTACGCCCTGGCGACGTTGTGACAGTGACCAGGCTAGACCGCCTGGCCCGCAGCACCCGCGACCTACTGGACATTGCCGAGCGCA
TCCAGGAGGCCGGCGCGGGCCTGCGTAGCCTGGCAGAGCCGTGGGCCGACACCACCACGCCGGCCGGCCGCATGGTGTTGACCGTGTTCGCCGGCATTGCCGAGTTCGA
GCGTTCCCTAATCATCGACCGCACCCGGAGCGGGCGCGAGGCCGCCAAGGCCCGAGGCGTGAAGTTTGGCCCCCGCCCTACCCTCACCCCGGCACAGATCGCGCACGCC
CGCGAGCTGATCGACCAGGAAGGCCGCACCGTGAAAGAGGCGGCTGCACTGCTTGGCGTGCATCGCTCGACCCTGTACCGCGCACTTGAGCGCAGCGAGGAAGTGACGC
CCACCGAGGCCAGGCGGCGCGGTGCCTTCCGTGAGGACGCATTGACCGAGGCCGACGCCCTGGCGGCCGCCGAGAATGAACGCCAAGAGGAACAAGCATGAAACCGCA
CCAGGACGGCCAGGACGAACCGTTTTTCATTACCGAAGAGATCGAGGCGGAGATGATCGCGGCCGGGTACGTGTTCGAGCCGCCCGCGCACGTCTCAACCGTGCGGCTG
CATGAAATCCTGGCCGGTTTGTCTGATGCCAAGCTGGCGGCCTGGCCGGCCAGCTTGGCCGCTGAAGAAACCGAGCGCCGCCGTCTAAAAAGGTGATGTGTATTTGAGT
AAAACAGCTTGCGTCATGCGGTCGCTGCGTATATGATGCGATGAGTAAATAAACAAATACGCAAGGGGAACGCATGAAGGTTATCGCTGTACTTAACCAGAAAGGCGGG
TCAGGCAAGACGACCATCGCAACCCATCTAGCCCGCGCCCTGCAACTCGCCGGGGCCGATGTTCTGTTAGTCGATTCCGATCCCCAGGGCAGTGCCCGCGATTGGGCGGC
CGTGCGGGAAGATCAACCGCTAACCGTTGTCGGCATCGACCGCCCGACGATTGACCGCGACGTGAAGGCCATCGGCCGGCGCGACTTCGTAGTGATCGACGGAGCGCCC
CAGGCGGCGGACTTGGCTGTGTCCGCGATCAAGGCAGCCGACTTCGTGCTGATTCCGGTGCAGCCAAGCCCTTACGACATATGGGCCACCGCCGACCTGGTGGAGCTGG
TTAAGCAGCGCATTGAGGTCACGGATGGAAGGCTACAAGCGGCCTTTGTCGTGTCGCGGGCGATCAAAGGCACGCGCATCGGCGGTGAGGTTGCCGAGGCGCTGGCCGG
GTACGAGCTGCCCATTCTTGAGTCCCGTATCACGCAGCGCGTGAGCTACCCAGGCACTGCCGCCGCCGGCACAACCGTTCTTGAATCAGAACCCGAGGGCGACGCTGCCC
GCGAGGTCCAGGCGCTGGCCGCTGAAATTAAATCAAAACTCATTTGAGTTAATGAGGTAAAGAGAAAATGAGCAAAAGCACAAACACGCTAAGTGCCGGCCGTCCGAG
CGCACGCAGCAGCAAGGCTGCAACGTTGGCCAGCCTGGCAGACACGCCAGCCATGAAGCGGGTCAACTTTCAGTTGCCGGCGGAGGATCACACCAAGCTGAAGATGTA
CGCGGTACGCCAAGGCAAGACCATTACCGAGCTGCTATCTGAATACATCGCGCAGCTACCAGAGTAAATGAGCAAATGAATAAATGAGTAGATGAATTTTAGCGGCTAA
AGGAGGCGGCATGGAAAATCAAGAACAACCAGGCACCGACGCCGTGGAATGCCCCATGTGTGGAGGAACGGGCGGTTGGCCAGGCGTAAGCGGCTGGGTTGTCTGCCG
GCCCTGCAATGGCACTGGAACCCCCAAGCCCGAGGAATCGGCGTGAGCGGTCGCAAACCATCCGGCCCGGTACAAATCGGCGCGGCGCTGGGTGATGACCTGGTGGAG
AAGTTGAAGGCCGCGCAGGCCGCCCAGCGGCAACGCATCGAGGCAGAAGCACGCCCCGGTGAATCGTGGCAAGCGGCCGCTGATCGAATCCGCAAAGAATCCCGGCAA
CCGCCGGCAGCCGGTGCGCCGTCGATTAGGAAGCCGCCCAAGGGCGACGAGCAACCAGATTTTTTCGTTCCGATGCTCTATGACGTGGGCACCCGCGATAGTCGCAGCA
TCATGGACGTGGCCGTTTTCCGTCTGTCGAAGCGTGACCGACGAGCTGGCGAGGTGATCCGCTACGAGCTTCCAGACGGGCACGTAGAGGTTTCCGCAGGGCCGGCCGG
CATGGCCAGTGTGTGGGATTACGACCTGGTACTGATGGCGGTTTCCCATCTAACCGAATCCATGAACCGATACCGGGAAGGGAAGGGAGACAAGCCCGGCCGCGTGTTC
CGTCCACACGTTGCGGACGTACTCAAGTTCTGCCGGCGAGCCGATGGCGGAAAGCAGAAAGACGACCTGGTAGAAACCTGCATTCGGTTAAACACCACGCACGTTGCCA
TGCAGCGTACGAAGAAGGCCAAGAACGGCCGCCTGGTGACGGTATCCGAGGGTGAAGCCTTGATTAGCCGCTACAAGATCGTAAAGAGCGAAACCGGGCGGCCGGAGT
ACATCGAGATCGAGCTAGCTGATTGGATGTACCGCGAGATCACAGAAGGCAAGAACCCGGACGTGCTGACGGTTCACCCCGATTACTTTTTGATCGATCCCGGCATCGG
CCGTTTTCTCTACCGCCTGGCACGCCGCGCCGCAGGCAAGGCAGAAGCCAGATGGTTGTTCAAGACGATCTACGAACGCAGTGGCAGCGCCGGAGAGTTCAAGAAGTTC
TGTTTCACCGTGCGCAAGCTGATCGGGTCAAATGACCTGCCGGAGTACGATTTGAAGGAGGAGGCGGGGCAGGCTGGCCCGATCCTAGTCATGCGCTACCGCAACCTGA
TCGAGGGCGAAGCATCCGCCGGTTCCTAATGTACGGAGCAGATGCTAGGGCAAATTGCCCTAGCAGGGGAAAAAGGTCGAAAAGGTCTCTTTCCTGTGGATAGCACGTA
CATTGGGAACCCAAAGCCGTACATTGGGAACCGGAACCCGTACATTGGGAACCCAAAGCCGTACATTGGGAACCGGTCACACATGTAAGTGACTGATATAAAAGAGAA
AAAAGGCGATTTTTCCGCCTAAAACTCTTTAAAACTTATTAAAACTCTTAAAACCCGCCTGGCCTGTGCATAACTGTCTGGCCAGCGCACAGCCGAAGAGCTGCAAAAAG
CGCCTACCCTTCGGTCGCTGCGCTCCCTACGCCCCGCCGCTTCGCGTCGGCCTATCGCGGCCGCTGGCCGCTCAAAAATGGCTGGCCTACGGCCAGGCAATCTACCAGGG
CGCGGACAAGCCGCGCCGTCGCCACTCGACCGCCGGCGCCCACATCAAGGCACCCTGCCTCGCGCGTTTCGGTGATGACGGTGAAAACCTCTGACACATGCAGCTCCCG
GAGACGGTCACAGCTTGTCTGTAAGCGGATGCCGGGAGCAGACAAGCCCGTCAGGGCGCGTCAGCGGGTGTTGGCGGGTGTCGGGGCGCAGCCATGACCCAGTCACGT
AGCGATAGCGGAGTGTATACTGGCTTAACTATGCGGCATCAGAGCAGATTGTACTGAGAGTGCACCATATGCGGTGTGAAATACCGCACAGATGCGTAAGGAGAAAATA
CCGCATCAGGCGCTCTTCCGCTTCCTCGCTCACTGACTCGCTGCGCTCGGTCGTTCGGCTGCGGCGAGCGGTATCAGCTCACTCAAAGGCGGTAATACGGTTATCCACAG
AATCAGGGGATAACGCAGGAAAGAACATGTGAGCAAAAGGCCAGCAAAAGGCCAGGAACCGTAAAAAGGCCGCGTTGCTGGCGTTTTTCCATAGGCTCCGCCCCCCTG
ACGAGCATCACAAAAATCGACGCTCAAGTCAGAGGTGGCGAAACCCGACAGGACTATAAAGATACCAGGCGTTTCCCCCTGGAAGCTCCCTCGTGCGCTCTCCTGTTCC
GACCCTGCCGCTTACCGGATACCTGTCCGCCTTTCTCCCTTCGGGAAGCGTGGCGCTTTCTCATAGCTCACGCTGTAGGTATCTCAGTTCGGTGTAGGTCGTTCGCTCCAA
GCTGGGCTGTGTGCACGAACCCCCCGTTCAGCCCGACCGCTGCGCCTTATCCGGTAACTATCGTCTTGAGTCCAACCCGGTAAGACACGACTTATCGCCACTGGCAGCAG
CCACTGGTAACAGGATTAGCAGAGCGAGGTATGTAGGCGGTGCTACAGAGTTCTTGAAGTGGTGGCCTAACTACGGCTACACTAGAAGGACAGTATTTGGTATCTGCGC
TCTGCTGAAGCCAGTTACCTTCGGAAAAAGAGTTGGTAGCTCTTGATCCGGCAAACAAACCACCGCTGGTAGCGGTGGTTTTTTTGTTTGCAAGCAGCAGATTACGCGCA
GAAAAAAAGGATCTCAAGAAGATCCTTTGATCTTTTCTACGGGGTCTGACGCTCAGTGGAACGAAAACTCACGTTAAGGGATTTTGGTCATGCATTCTAGGTACTAAAAC
AATTCATCCAGTAAAATATAATATTTTATTTTCTCCCAATCAGGCTTGATCCCCAGTAAGTCAAAAAATAGCTCGACATACTGTTCTTCCCCGATATCCTCCCTGATCGAC
CGGACGCAGAAGGCAATGTCATACCACTTGTCCGCCCTGCCGCTTCTCCCAAGATCAATAAAGCCACTTACTTTGCCATCTTTCACAAAGATGTTGCTGTCTCCCAGGTCG
CCGTGGGAAAAGACAAGTTCCTCTTCGGGCTTTTCCGTCTTTAAAAAATCATACAGCTCGCGCGGATCTTTAAATGGAGTGTCTTCTTCCCAGTTTTCGCAATCCACATCG
GCCAGATCGTTATTCAGTAAGTAATCCAATTCGGCTAAGCGGCTGTCTAAGCTATTCGTATAGGGACAATCCGATATGTCGATGGAGTGAAAGAGCCTGATGCACTCCGC
ATACAGCTCGATAATCTTTTCAGGGCTTTGTTCATCTTCATACTCTTCCGAGCAAAGGACGCCATCGGCCTCACTCATGAGCAGATTGCTCCAGCCATCATGCCGTTCAAA
GTGCAGGACCTTTGGAACAGGCAGCTTTCCTTCCAGCCATAGCATCATGTCCTTTTCCCGTTCCACATCATAGGTGGTCCCTTTATACCGGCTGTCCGTCATTTTTAAATAT
AGGTTTTCATTTTCTCCCACCAGCTTATATACCTTAGCAGGAGACATTCCTTCCGTATCTTTTACGCAGCGGTATTTTTCGATCAGTTTTTCAATTCCGGTGATATTCTCAT
TTTAGCCATTTATTATTTCCTTCCTCTTTTCTACAGTATTTAAAGATACCCCAAGAAGCTAATTATAACAAGACGAACTCCAATTCACTGTTCCTTGCATTCTAAAACCTTA
AATACCAGAAAACAGCTTTTTCAAAGTTGTTTTCAAAGTTGGCGTATAACATAGTATCGACGGAGCCGATTTTGAAACCGCGGTGATCACAGGCAGCAACGCTCTGTCAT
CGTTACAATCAACATGCTACCCTCCGCGAGATCATCCGTGTTTCAAACCCGGCAGCTTAGTTGCCGTTCTTCCGAATAGCATCGGTAACATGAGCAAAGTCTGCCGCCTTA

CAACGGCTCTCCCGCTGACGCCGTCCCGGACTGATGGGCTGCCTGTATCGAGTGGTGATTTTGTGCCGAGCTGCCGGTCGGGGAGCTGTTGGCTGGCTGGTGGCAGGATA
TATTGTGGTGTAAACAAATTGACGCTTAGACAACTTAATAACACATTGCGGACGTTTTTAATGTACTGAATTAACGCCGAATTAATTCGGGGGATCTGGATTTTAGTACTG
GATTTTGGTTTTAGGAATTAGAAATTTTATTGATAGAAGTATTTTACAAATACAAATACATACTAAGGGTTTCTTATATGCTCAACACATGAGCGAAACCCTATAGGAACC
CTAATTCCCTTATCTGGGAACTACTCACACATTATTATGGAGAAACTCGAGCTTGTCGATCGACAGATCCGGTCGGCATCTACTCTATTTCTTTGCCCTCGGACGAGTGCT
GGGGCGTCGGTTTCCACTATCGGCGAGTACTTCTACACAGCCATCGGTCCAGACGGCCGCGCTTCTGCGGGCGATTTGTGTACGCCCGACAGTCCCGGCTCCGGATCGGA
CGATTGCGTCGCATCGACCCTGCGCCCAAGCTGCATCATCGAAATTGCCGTCAACCAAGCTCTGATAGAGTTGGTCAAGACCAATGCGGAGCATATACGCCCGGAGTCGT
GGCGATCCTGCAAGCTCCGGATGCCTCCGCTCGAAGTAGCGCGTCTGCTGCTCCATACAAGCCAACCACGGCCTCCAGAAGAAGATGTTGGCGACCTCGTATTGGGAATC
CCCGAACATCGCCTCGCTCCAGTCAATGACCGCTGTTATGCGGCCATTGTCCGTCAGGACATTGTTGGAGCCGAAATCCGCGTGCACGAGGTGCCGGACTTCGGGGCAGT
CCTCGGCCCAAAGCATCAGCTCATCGAGAGCCTGCGCGACGGACGCACTGACGGTGTCGTCCATCACAGTTTGCCAGTGATACACATGGGGATCAGCAATCGCGCATAT
GAAATCACGCCATGTAGTGTATTGACCGATTCCTTGCGGTCCGAATGGGCCGAACCCGCTCGTCTGGCTAAGATCGGCCGCAGCGATCGCATCCATAGCCTCCGCGACCG
GTTGTAGAACAGCGGGCAGTTCGGTTTCAGGCAGGTCTTGCAACGTGACACCCTGTGCACGGCGGGAGATGCAATAGGTCAGGCTCTCGCTAAACTCCCCAATGTCAAG
CACTTCCGGAATCGGGAGCGCGGCCGATGCAAAGTGCCGATAAACATAACGATCTTTGTAGAAACCATCGGCGCAGCTATTTACCCGCAGGACATATCCACGCCCTCCT
ACATCGAAGCTGAAAGCACGAGATTCTTCGCCCTCCGAGAGCTGCATCAGGTCGGAGACGCTGTCGAACTTTTCGATCAGAAACTTCTCGACAGACGTCGCGGTGAGTTC
AGGCTTTTTCATATCTCATTGCCCCCCGGGATCTGCGAAAGCTCGAGAGAGATAGATTTGTAGAGAGAGACTGGTGATTTCAGCGTGTCCTCTCCAAATGAAATGAACTT
CCTTATATAGAGGAAGGTCTTGCGAAGGATAGTGGGATTGTGCGTCATCCCTTACGTCAGTGGAGATATCACATCAATCCACTTGCTTTGAAGACGTGGTTGGAACGTCT
TCTTTTTCCACGATGCTCCTCGTGGGTGGGGGTCCATCTTTGGGACCACTGTCGGCAGAGGCATCTTGAACGATAGCCTTTCCTTTATCGCAATGATGGCATTTGTAGGTG
CCACCTTCCTTTTCTACTGTCCTTTTGATGAAGTGACAGATAGCTGGGCAATGGAATCCGAGGAGGTTTCCCGATATTACCCTTTGTTGAAAAGTCTCAATAGCCCTTTGG
TCTTCTGAGACTGTATCTTTGATATTCTTGGAGTAGACGAGAGTGTCGTGCTCCACCATGTTATCACATCAATCCACTTGCTTTGAAGACGTGGTTGGAACGTCTTCTTTTT
CCACGATGCTCCTCGTGGGTGGGGGTCCATCTTTGGGACCACTGTCGGCAGAGGCATCTTGAACGATAGCCTTTCCTTTATCGCAATGATGGCATTTGTAGGTGCCACCTT
CCTTTTCTACTGTCCTTTTGATGAAGTGACAGATAGCTGGGCAATGGAATCCGAGGAGGTTTCCCGATATTACCCTTTGTTGAAAAGTCTCAATAGCCCTTTGGTCTTCTG
AGACTGTATCTTTGATATTCTTGGAGTAGACGAGAGTGTCGTGCTCCACCATGTTGGCAAGCTGCTCTAGCCAATACGCAAACCGCCTCTCCCCGCGCGTTGGCCGATTCA
TTAATGCAGCTGGCACGACAGGTTTCCCGACTGGAAAGCGGGCAGTGAGCGCAACGCAATTAATGTGAGTTAGCTCACTCATTAGGCACCCCAGGCTTTACACTTTATGC
TTCCGGCTCGTATGTTGTGTGGAATTGTGAGCGGATAACAATTTCACACAGGAAACAGCTATGACCATGATT

**SpCas12f1:** The *SpCas12f1* protein gene (Bigelyte et al. 2021), underlined, was inserted between the marked restriction enzyme sequence in pIZZA-BYR to form pIZZA-BYR-SpCas12f1. The HiBiT sequence is marked in yellow, the SV40 nuclear localization signal is marked in blue, and the nucleoplasmin nuclear localization signal is marked in green.

ATGGTGAGCGGCTGGCGGCTGTTCAAGAAGATTTCCGGGTCAGGGCCAAAGAAGAAGCGGAAGGTCGGGTCAGGGGGCGAAAGCGTGAAAGCAATTAAGCTGAAGATC
CTGGATATGTTTCTGGACCCCGAGTGCACGAAGCAGGATGATAACTGGCGCAAAGATTTGTCTACTATGTCCAGATTCTGCGCTGAGGCCGGGAATATGTGCCTGCGGGA
CCTGTATAATTACTTTAGCATGCCCAAGGAAGATCGGATTTCCAGCAAAGACTTATACAACGCCATGTATCATAAAACTAAACTTCTCCACCCAGAGCTGCCAGGTAAGG
TCGCGAATCAAATCGTGAACCACGCTAAGGATGTTTGGAAACGCAACGCTAAACTCATTTATCGGAATCAAATCTCAATGCCTACTTATAAGATCACAACCGCCCCAATC
CGGCTGCAGAATAACATTTATAAGTTAATCAAGAATAAGAACAAGTACATCATAGACGTGCAGCTGTACTCCAAGGAATACTCCAAGGATAGTGGTAAGGGCACTCATA
GGTATTTCCTGGTCGCCGTTAGAGACTCATCTACCCGGATGATATTCGATAGGATTATGAGTAAGGACCATATTGACAGTTCTAAGTCATACACGCAGGGACAGCTCCAG
ATCAAGAAAGACCACCAGGGGAAGTGGTATTGCATCATCCCCTATACTTTCCCTACACATGAGACAGTCCTCGACCCGGATAAAGTCATGGGAGTCGACCTTGGCGTGG
CCAAAGCTGTGTACTGGGCGTTCAATAGCTCTTACAAAAGAGGCTGTATCGATGGAGGGGAGATCGAGCATTTCCGCAAAATGATACGAGCACGAAGAGTGAGCATCCA
AAATCAGATCAAGCACTCAGGAGACGCCCGTAAAGGACATGGGCGCAAAAGGGCCCTAAAACCCATAGAAACATTGAGCGAGAAGGAGAAGAATTTTAGGGATACCAT
AAACCACAGATATGCAAATCGAATTGTAGAAGCCGCTATTAAGCAGGGCTGTGGGACGATCCAGATCGAGAACCTTGAGGGTATAGCTGACACCACAGGCAGTAAATTT
CTCAAGAACTGGCCTTACTACGACCTGCAGACAAAGATTGTGAATAAAGCCAAGGAACACGGCATTACCGTGGTTGCAATCAACCCCCAATATACATCCCAAAGGTGCT
CGATGTGCGGGTATATTGAAAAAACCAACCGTTCTAGCCAGGCAGTGTTTGAGTGTAAGCAGTGCGGTTACGGCAGTCGGACTATATGTATTAACTGCAGGCACGTCCA
AGTATCCGGGGATGTTTGTGAGGAATGTGGCGGCATCGTAAAAAAAGAAAACGTGAACGCAGACTACAATGCGGCCAAGAACATTTCCACCCCGTACATCGACCAGATA
ATCATGGAGAAGTGTCTGGAACTAGGGATTCCTTACCGCAGCATCACCTGTAAGGAATGTGGTCACATCCAGGCTTCAGGAAATACCTGCGAGGTGTGCGGATCTACTA
ATATTTTGAAGCCAAAGAAAATTAGAAAAGCCAAAAAAAGGCCGGCGGCCACGAAAAAGGCCGGCCAGGCAAAAAAGAAAAAGTAA

**CasMINIv3.1:** The *CasMINIv3.1* protein gene (Xu et al. 2021), underlined, was inserted between the marked restriction enzyme sequence in pIZZA-BYR to form pIZZA-BYR-CasMINIv3.1. The HiBiT sequence is marked in yellow, the SV40 nuclear localisation signal is marked in blue, and the nucleoplasmin nuclear localisation signal is marked in green.

ATGGTGAGCGGCTGGCGGCTGTTCAAGAAGATTTCCGGGTCAGGGCCAAAGAAGAAGCGGAAGGTCGGGTCAGGGGCCAAAAACACCATTACCAAAACACTGAAACTG
CGTATTGTGCGTCCGTATAATAGCGCAGAAGTGGAAAAAATTGTTGCCGACGAAAAAAACAACCGCGAAAAAATCGCACTGGAAAAGAACAAAGACAAAGTGAAAGA
AGCCTGCAGCAAACATCTGAAAGTTGCAGCATATTGTACCACACAGGTTGAACGTAATGCATGCCTGTTTTGTAAAGCACGTAAACTGGATGACAAATTCTACCAAAAA
CTGCGTGGTCAGTTTCCGGATGCAGTTTTTTGGCAAGAAATCAGCGAAATTTTTCGCCAGCTGCAGAAACAGGCAGCAGAAATCTATAATCAGAGCCTGATCGAACTGTA
CTACGAGATTTTTATCAAAGGCAAAGGTATTGCAAATGCCAGCAGCGTTGAACATTATCTGAGTAGAGTTTGTTATAGACGTGCAGCAGCTCTGTTTAAAAACGCAGCAA
TTGCAAGCGGTCTGCGTAGCAAAATCAAAAGCAATTTTCGTCTGAAAGAACTGAAAAACATGAAAAGTGGTCTGCCGACCACCAAAAGCGATAATTTTCCGATTCCGCT
GGTTAAACAGAAAGGTGGTCAGTATACCGGTTTTGAAATTAGCAATCATAATAGCGACTTCATCATCAAGATTCCGTTTGGTCGTTGGCAGGTCAAAAAAGAGATTGATA
AATATCGTCCGTGGGAGAAATTTGACTTTGAACAGGTTCAGAAAAGCCCGAAACCGATTAGCCTGCTGCTGAGCACCCAGCGTCGTAAACGTAATAAAGGTTGGAGCAA
AGATGAAGGCACCGAAGCCGAAATCAAAAAAGTTATGAATGGCGATTATCAGACCAGCTACATTGAAGTTAAACGTGGCAGCAAAATCTGTGAAAAAAGCGCATGGAT
GCTGAATCTGAGCATTGATGTTCCGAAAATTGATAAAGGTGTGGATCCGAGCATTATTGGTGGTATTGATGTTGGTGTTAAATCACCGCTGGTTTGCGCAATTAACAATG
CATTTAGCCGTTATAGCATCAGCGATAACGACCTGTTTCACTTCAACAAGAAAATGTTTGCACGTCGTCGTATCCTGCTGAAAAAAAACCGTCATAAACGTGCAGGTCAT
GGTGCAAAAAACAAACTGAAACCGATCACCATTCTGACCGAAAAAAGTGAACGTTTTCGCAAAAAGCTGATTGAACGTTGGGCATGTGAAATCGCGGATTTCTTCATTA
AAAACAAAGTTGGCACCGTGCAGATGGAAAATCTGGAAAGCATGAAACGTAAAGAGGACAGCTATTTTAACATTCGCCTGCGTGGCTTTTGGCCGTATGCAGAAATGCA
GAACAAAATCGAATTCAAACTGAAGCAGTATGGCATCGAAATTCGTAAAGTTGCACCGAATAATACCAGCAAAACCTGTAGCAAATGTGGCCATCTGAACAACTATTTC
AACTTCGAGTACCGCAAGAAAAACAAATTCCCGCACTTTAAATGCGAAAAATGCAACTTCAAAGAAAACGCCGATTATAATGCAGCCCTGAATATTTCAAACCCGAAAC
TGAAAAGCACCAAAGAGGAACCGAAAAGGCCGGCGGCCACGAAAAAGGCCGGCCAGGCAAAAAAGAAAAAGTAA

**AsCas12f1:** The *AsCas12f1* protein gene (Wu et al. 2021), underlined, was inserted between the marked restriction enzyme sequence in pIZZA-BYR to form pIZZA-BYR-AsCas12f. The HiBiT sequence is marked in yellow, the SV40 nuclear localization signal is marked in blue, and the nucleoplasmin nuclear localization signal is marked in green.

ATGGTGAGCGGCTGGCGGCTGTTCAAGAAGATTTCCGGGTCAGGGCCAAAGAAGAAGCGGAAGGTCGGGTCAGGGATCAAGGTGTACAGATACGAGATCGTGAAGCCT
CTGGACCTGGACTGGAAGGAGTTCGGCACCATCCTGAGACAGCTGCAGCAGGAAACCAGATTCGCCCTGAATAAGGCCACACAGCTGGCCTGGGAGTGGATGGGCTTCA
GCAGCGACTACAAGGATAACCACGGCGAGTACCCCAAGAGCAAGGACATCCTGGGCTACACCAACGTGCACGGCTACGCCTACCACACCATCAAGACAAAGGCCTACA
GACTGAACTCTGGAAATCTGAGCCAGACCATCAAGAGAGCCACAGACAGGTTCAAGGCCTACCAGAAGGAGATCCTGCGCGGCGACATGTCTATCCCCAGCTACAAGA
GGGACATCCCCCTGGACCTGATCAAGGAGAACATCTCCGTGAACAGGATGAATCACGGCGACTACATCGCCAGCCTGTCTCTGCTGAGCAACCCCGCCAAGCAGGAGAT
GAACGTGAAGAGAAAGATCTCCGTGATCATCATCGTGAGGGGCGCCGGCAAGACCATCATGGACAGAATCCTGTCCGGCGAGTACCAGGTGAGCGCCAGCCAGATTATC
CACGACGACCGGAAGAACAAGTGGTACCTGAACATCAGCTACGACTTCGAGCCACAGACCAGAGTGCTGGACCTGAACAAGATCATGGGCATTGACCTGGGCGTGGCC
GTGGCCGTGTACATGGCCTTCCAGCACACCCCCGCCAGGTACAAGCTGGAGGGCGGCGAGATTGAGAACTTCAGGAGGCAGGTGGAGAGCCGGCGCATCTCCATGCTGA
GACAGGGCAAGTACGCCGGCGGCGCCAGGGGCGGCCACGGCAGAGACAAGAGAATCAAGCCCATTGAGCAGCTGAGGGATAAGATCGCCAATTTCAGAGACACCACC
AATCACCGGTACAGCAGATACATCGTGGACATGGCCATCAAGGAGGGCTGCGGCACAATCCAGATGGAGGATCTGACAAACATCAGAGACATCGGCAGCAGATTCCTG
CAGAACTGGACCTACTACGACCTGCAGCAGAAGATCATCTACAAGGCCGAGGAGGCCGGCATCAAAGTGATCAAGATCGACCCCCAGTACACCAGCCAGAGATGCTCC

GAGTGCGGCAACATCGACTCCGGCAACAGAATCGGCCAGGCCATCTTTAAGTGCCGGGCCTGCGGCTACGAGGCCAACGCCGACTACAACGCCGCCCGGAATATCGCCA
TCCCCAACATCGACAAGATCATCGCCGAGAGCATTAAGAAAAGGCCGGCGGCCACGAAAAAGGCCGGCCAGGCAAAAAAGAAAAAGTAA

**Cas12j-2:** The *Cas12j-2* protein gene (Pausch et al. 2020), underlined, was inserted between the marked restriction enzyme sequence in pIZZA-BYR to form pIZZA-BYR-Cas12j-2. The HiBiT sequence is marked in yellow, the 2xSV40 nuclear localisation signal is marked in pink.

ATGCCAAAACCCGCGGTTGAGAGTGAATTCTCGAAAGTGCTTAAGAAGCACTTCCCCGGGGAGCGCTTCCGTTCCAGTTACATGAAACGCGGTGGCAAGATCTTAGCCG
CCCAGGGAGAAGAGGCCGTAGTTGCATACTTACAGGGAAAATCAGAGGAGGAACCGCCTAACTTCCAACCTCCCGCGAAATGTCATGTGGTTACTAAAAGTCGCGATTT
TGCCGAATGGCCGATTATGAAAGCCAGCGAGGCTATTCAACGCTACATCTACGCGTTGTCAACCACTGAGCGTGCCGCGTGTAAACCAGGGAAAAGCAGCGAATCACAT
GCTGCCTGGTTTGCAGCGACTGGCGTCAGTAACCACGGGTATTCACATGTCCAAGGGCTTAACTTAATCTTTGACCATACTCTGGGGCGTTACGATGGGGTTTTGAAGAA
GGTTCAACTGCGTAATGAAAAGGCACGTGCCCGTTTGGAGTCAATTAATGCATCTCGTGCCGATGAGGGTTTACCTGAAATCAAGGCGGAAGAGGAAGAGGTAGCCACA
AATGAGACAGGGCATTTGCTTCAACCGCCAGGCATTAATCCCAGCTTCTATGTTTATCAAACGATCTCACCTCAGGCTTATCGTCCACGCGACGAGATTGTTTTGCCCCCC
GAGTATGCAGGGTACGTGCGTGATCCCAACGCCCCCATTCCGTTAGGAGTAGTCCGCAACCGTTGTGATATTCAGAAAGGCTGTCCGGGCTATATTCCTGAGTGGCAGCG
CGAGGCAGGTACTGCGATCTCTCCGAAGACTGGGAAAGCCGTTACAGTTCCTGGCCTTTCCCCTAAGAAGAACAAGCGTATGCGCCGCTATTGGCGTTCGGAAAAAGAG
AAGGCACAAGATGCACTGTTGGTTACCGTCCGTATCGGTACTGACTGGGTAGTGATTGATGTGCGCGGATTATTGCGTAATGCCCGCTGGCGCACCATTGCTCCAAAGGA
CATCTCATTAAACGCGCTTTTAGACCTGTTCACTGGAGACCCAGTGATTGATGTCCGCCGCAACATTGTAACTTTTACCTACACGCTTGATGCCTGCGGTACCTATGCTCG
TAAATGGACGTTAAAAGGAAAGCAGACAAAGGCAACACTGGATAAACTGACTGCCACCCAAACAGTAGCGCTGGTAGCGATCGATTTGGGCCAGACGAACCCTATTTC
AGCGGGAATCTCCCGTGTTACGCAGGAAAACGGGGCGCTTCAATGTGAGCCGCTGGACCGCTTTACGTTACCTGACGACTTGTTGAAAGATATCAGTGCGTACCGCATCG
CATGGGACCGCAACGAGGAGGAATTACGTGCCCGTTCAGTCGAAGCGTTACCCGAAGCACAGCAGGCCGAAGTACGTGCTCTGGACGGCGTAAGCAAGGAGACCGCTC
GTACTCAATTATGTGCTGATTTCGGCCTTGATCCCAAGCGTCTGCCTTGGGACAAGATGTCCTCAAACACTACATTCATCAGCGAAGCCCTGTTGTCAAATAGTGTATCGC
GTGACCAGGTTTTCTTTACCCCAGCTCCTAAAAAAGGAGCCAAAAAAAAAGCTCCGGTGGAGGTTATGCGCAAGGACCGTACTTGGGCCCGTGCTTATAAACCACGTCT
GTCTGTTGAAGCCCAGAAACTGAAAAACGAAGCTCTGTGGGCCCTTAAACGTACGAGTCCCGAATACCTTAAATTAAGCCGTCGTAAAGAGGAGCTTTGCCGTCGCAGT
ATCAATTACGTTATCGAGAAGACCCGCCGTCGTACCCAGTGTCAAATCGTGATCCCGGTGATTGAAGATCTGAACGTCCGCTTTTTCCACGGATCTGGGAAACGTCTGCC
TGGCTGGGATAATTTCTTTACAGCGAAAAAAGAGAACCGTTGGTTCATCCAGGGCCTTCACAAGGCTTTTTCCGACTTACGCACACATCGCAGTTTCTACGTTTTCGAGGT
ACGTCCAGAGCGCACCTCCATTACCTGCCCCAAATGCGGGCACTGCGAGGTTGGGAACCGTGATGGCGAAGCGTTCCAATGCCTTTCCTGTGGAAAAACATGTAATGCA
GACTTAGACGTAGCCACTCACAACCTGACACAGGTTGCCCTTACCGGAAAAACCATGCCTAAACGTGAAGAACCACGCGATGCCCAGGGGACCGCTCCGGCACGTAAGA
CCAAAAAAGCATCAAAGTCGAAGGCCCCCCTGCTGAACGCGAGGACCAGACCCCGGCCCAAGAACCTAGCCAGACCAGCGGGTCAGGGCCTAAGAAGAAGCGTAAAG
TAGAAGATCCTAAGAAAAAACGCAAGGTGGGCAGCAGCGGAGTGAGCGGCTGGCGGCTGTTCAAGAAGATTTCCTAA

**SpCas9:** The human codon-optimized *SpCas9* protein gene, underlined, was inserted between the marked nucleotides in pIZZA-BYR to form pIZZA-BYR-SpCas9. The SV40 nuclear localization signal is marked in blue, and the nucleoplasmin nuclear localization signal is marked in green.

ATGGCCCCAAAGAAGAAGCGGAAGGTCGGTATCCACGGAGTCCCAGCAGCCGACAAGAAGTACAGCATCGGCCTGGACATCGGCACCAACTCTGTGGGCTGGGCCGTG
ATCACCGACGAGTACAAGGTGCCCAGCAAGAAATTCAAGGTGCTGGGCAACACCGACCGGCACAGCATCAAGAAGAACCTGATCGGAGCCCTGCTGTTCGACAGCGGC
GAAACAGCCGAGGCCACCCGGCTGAAGAGAACCGCCAGAAGAAGATACACCAGACGGAAGAACCGGATCTGCTATCTGCAAGAGATCTTCAGCAACGAGATGGCCAAG
GTGGACGACAGCTTCTTCCACAGACTGGAAGAGTCCTTCCTGGTGGAAGAGGATAAGAAGCACGAGCGGCACCCCATCTTCGGCAACATCGTGGACGAGGTGGCCTACC
ACGAGAAGTACCCCACCATCTACCACCTGAGAAAGAAACTGGTGGACAGCACCGACAAGGCCGACCTGCGGCTGATCTATCTGGCCCTGGCCCACATGATCAAGTTCCG
GGGCCACTTCCTGATCGAGGGCGACCTGAACCCCGACAACAGCGACGTGGACAAGCTGTTCATCCAGCTGGTGCAGACCTACAACCAGCTGTTCGAGGAAAACCCCATC
AACGCCAGCGGCGTGGACGCCAAGGCCATCCTGTCTGCCAGACTGAGCAAGAGCAGACGGCTGGAAAATCTGATCGCCCAGCTGCCCGGCGAGAAGAAGAATGGCCTG
TTCGGAAACCTGATTGCCCTGAGCCTGGGCCTGACCCCCAACTTCAAGAGCAACTTCGACCTGGCCGAGGATGCCAAACTGCAGCTGAGCAAGGACACCTACGACGACG
ACCTGGACAACCTGCTGGCCCAGATCGGCGACCAGTACGCCGACCTGTTTCTGGCCGCCAAGAACCTGTCCGACGCCATCCTGCTGAGCGACATCCTGAGAGTGAACAC
CGAGATCACCAAGGCCCCCCTGAGCGCCTCTATGATCAAGAGATACGACGAGCACCACCAGGACCTGACCCTGCTGAAAGCTCTCGTGCGGCAGCAGCTGCCTGAGAAG
TACAAAGAGATTTTCTTCGACCAGAGCAAGAACGGCTACGCCGGCTACATTGACGGCGGAGCCAGCCAGGAAGAGTTCTACAAGTTCATCAAGCCCATCCTGGAAAAGA
TGGACGGCACCGAGGAACTGCTCGTGAAGCTGAACAGAGAGGACCTGCTGCGGAAGCAGCGGACCTTCGACAACGGCAGCATCCCCCACCAGATCCACCTGGGAGAGC
TGCACGCCATTCTGCGGCGGCAGGAAGATTTTTACCCATTCCTGAAGGACAACCGGGAAAAGATCGAGAAGATCCTGACCTTCCGCATCCCCTACTACGTGGGCCCTCTG
GCCAGGGGAAACAGCAGATTCGCCTGGATGACCAGAAAGAGCGAGGAAACCATCACCCCCTGGAACTTCGAGGAAGTGGTGGACAAGGGCGCTTCCGCCCAGAGCTTC
ATCGAGCGGATGACCAACTTCGATAAGAACCTGCCCAACGAGAAGGTGCTGCCCAAGCACAGCCTGCTGTACGAGTACTTCACCGTGTATAACGAGCTGACCAAAGTGA
AATACGTGACCGAGGGAATGAGAAAGCCCGCCTTCCTGAGCGGCGAGCAGAAAAAGGCCATCGTGGACCTGCTGTTCAAGACCAACCGGAAAGTGACCGTGAAGCAGC
TGAAAGAGGACTACTTCAAGAAAATCGAGTGCTTCGACTCCGTGGAAATCTCCGGCGTGGAAGATCGGTTCAACGCCTCCCTGGGCACATACCACGATCTGCTGAAAAT
TATCAAGGACAAGGACTTCCTGGACAATGAGGAAAACGAGGACATTCTGGAAGATATCGTGCTGACCCTGACACTGTTTGAGGACAGAGAGATGATCGAGGAACGGCT
GAAAACCTATGCCCACCTGTTCGACGACAAAGTGATGAAGCAGCTGAAGCGGCGGAGATACACCGGCTGGGGCAGGCTGAGCCGGAAGCTGATCAACGGCATCCGGGA
CAAGCAGTCCGGCAAGACAATCCTGGATTTCCTGAAGTCCGACGGCTTCGCCAACAGAAACTTCATGCAGCTGATCCACGACGACAGCCTGACCTTTAAAGAGGACATC
CAGAAAGCCCAGGTGTCCGGCCAGGGCGATAGCCTGCACGAGCACATTGCCAATCTGGCCGGCAGCCCCGCCATTAAGAAGGGCATCCTGCAGACAGTGAAGGTGGTG
GACGAGCTCGTGAAAGTGATGGGCCGGCACAAGCCCGAGAACATCGTGATCGAAATGGCCAGAGAGAACCAGACCACCCAGAAGGGACAGAAGAACAGCCGCGAGAG
AATGAAGCGGATCGAAGAGGGCATCAAAGAGCTGGGCAGCCAGATCCTGAAAGAACACCCCGTGGAAAACACCCAGCTGCAGAACGAGAAGCTGTACCTGTACTACCT
GCAGAATGGGCGGGATATGTACGTGGACCAGGAACTGGACATCAACCGGCTGTCCGACTACGATGTGGACCATATCGTGCCTCAGAGCTTTCTGAAGGACGACTCCATC
GACAACAAGGTGCTGACCAGAAGCGACAAGAACCGGGGCAAGAGCGACAACGTGCCCTCCGAAGAGGTCGTGAAGAAGATGAAGAACTACTGGCGGCAGCTGCTGAA
CGCCAAGCTGATTACCCAGAGAAAGTTCGACAATCTGACCAAGGCCGAGAGAGGCGGCCTGAGCGAACTGGATAAGGCCGGCTTCATCAAGAGACAGCTGGTGGAAAC
CCGGCAGATCACAAAGCACGTGGCACAGATCCTGGACTCCCGGATGAACACTAAGTACGACGAGAATGACAAGCTGATCCGGGAAGTGAAAGTGATCACCCTGAAGTC
CAAGCTGGTGTCCGATTTCCGGAAGGATTTCCAGTTTTACAAAGTGCGCGAGATCAACAACTACCACCACGCCCACGACGCCTACCTGAACGCCGTCGTGGGAACCGCCC
TGATCAAAAAGTACCCTAAGCTGGAAAGCGAGTTCGTGTACGGCGACTACAAGGTGTACGACGTGCGGAAGATGATCGCCAAGAGCGAGCAGGAAATCGGCAAGGCTA
CCGCCAAGTACTTCTTCTACAGCAACATCATGAACTTTTTCAAGACCGAGATTACCCTGGCCAACGGCGAGATCCGGAAGCGGCCTCTGATCGAGACAAACGGCGAAAC
CGGGGAGATCGTGTGGGATAAGGGCCGGGATTTTGCCACCGTGCGGAAAGTGCTGAGCATGCCCCAAGTGAATATCGTGAAAAAGACCGAGGTGCAGACAGGCGGCTT
CAGCAAAGAGTCTATCCTGCCCAAGAGGAACAGCGATAAGCTGATCGCCAGAAAGAAGGACTGGGACCCTAAGAAGTACGGCGGCTTCGACAGCCCCACCGTGGCCTA
TTCTGTGCTGGTGGTGGCCAAAGTGGAAAAGGGCAAGTCCAAGAAACTGAAGAGTGTGAAAGAGCTGCTGGGGATCACCATCATGGAAAGAAGCAGCTTCGAGAAGAA
TCCCATCGACTTTCTGGAAGCCAAGGGCTACAAAGAAGTGAAAAAGGACCTGATCATCAAGCTGCCTAAGTACTCCCTGTTCGAGCTGGAAAACGGCCGGAAGAGAATG
CTGGCCTCTGCCGGCGAACTGCAGAAGGGAAACGAACTGGCCCTGCCCTCCAAATATGTGAACTTCCTGTACCTGGCCAGCCACTATGAGAAGCTGAAGGGCTCCCCCG
AGGATAATGAGCAGAAACAGCTGTTTGTGGAACAGCACAAGCACTACCTGGACGAGATCATCGAGCAGATCAGCGAGTTCTCCAAGAGAGTGATCCTGGCCGACGCTA
ATCTGGACAAAGTGCTGTCCGCCTACAACAAGCACCGGGATAAGCCCATCAGAGAGCAGGCCGAGAATATCATCCACCTGTTTACCCTGACCAATCTGGGAGCCCCTGC
CGCCTTCAAGTACTTTGACACCACCATCGACCGGAAGAGGTACACCAGCACCAAAGAGGTGCTGGACGCCACCCTGATCCACCAGAGCATCACCGGCCTGTACGAGACA
CGGATCGACCTGTCTCAGCTGGGAGGCGACAAAAGGCCGGCGGCCACGAAAAAGGCCGGCCAGGCAAAAAAGAAAAAGTAACTAGAGCGGCCGCATGCGTTC

**pBYR2eFa-U6:** Geminiviral replicon-based vector with an *Arabidopsis* U6-26 promoter for cloning of Cas12j-2, SpCas12f1, AsCas12f1, CasMINIv3.1 and SpCas9 gRNA scaffolds. The *p19* gene is marked in gold. The geminiviral long intergenic region (LIR) is marked in yellow, the geminiviral short intergenic region (SIR) is marked in grey, and the C1/C2 gene is marked in pink. The U6-26 promoter is marked in red. BsaI restriction enzyme sites for cloning the gRNA scaffolds are underlined and enlarged.

CGATCGCCGATCTAGTAACATAGATGACACCGCGCGATAATTTATCCTAGTTTGCGCGCTATATTTTGTTTTCTATCGCGTATTAAATGTATAATTGCGGGACTCTAAT
CATAAAAACCCATCTCATAAATAACGTCATGCATTACATGTTAATTATTACATGCTTAACGTAATTCAACAGAAATTATATGATAATCATCGCAAGACCGGCAACAGGAT

TCAATCTTAAGAAACTTTATTGCCAAATGTTTGAACGATCTGCTTACTCGCCTTCTTTTTCGAAGGTTTGAGTACCTTCAGGGCATCCTCTTGATACATTACTTTCCACTTC
GATTGGGGCAAGCTGTAGCAGTTCTTGCTTAGACCGAATTGCCATCTCACAGAGATGCTGAAGAGTTCGCGACCCTCCAGAAACGGTGATACTAACTCCTCGAAACCGA
ATACTATAGGTACATCCGATCTGGTCGAAACCGAAAAATCGAGATGCTGCATAGTTAACCGAATCTCCCGTCCAAGATCCAAGGACTCTGTGCAGTGAAGCTTCCGTCCT
GTCGTATCTGAGATATCTCTTAAATACAACTTTCCCGAAACCCCAGCTTTCCTTGAAACCAAGGGGATTATCTTGATTCGAATTCGTCTCATCGTTATGTAGCCGCCACTC
AGTCCAACTCGGACTTTCGTCAGGAAGTTTGAAGGGAGAAGTTGTACCTCCTGATCCTCCATCCCAACGTTCACTGTTAGCTTGTTCCCTAGCGTCGTTTCCTTGTATAGC
TCGTTCCATGGCTATCGTTCGTAAATGGTGAAAATTTTCAGAAAATTGCTTTTGCTTTAAAAGAAATGATTTAAATTGCTGCAATAGAAGTAGAATGCTTGATTGCTTGAG
ATTCGTTTGTTTTGTATATGTTGTGTTGAGAATTAATTCCCCTCGACTAGAGTCGAGATCTGGATTGAGAGTGAATATGAGACTCTAATTGGATACCGAGGGGAATTTATG
GAACGTCAGTGGAGCATTTTTGACAAGAAATATTTGCTAGCTGATACTGACCTTACGCGACTTTTGAACGCGCAATAATGGATTCTGACGTATGTGCTTAGCTCATTAAA
CTCCAGAAACCCGCGGCTGAGTGGCTCCTTCAACGTTGCGGTTCTGTCAGTTCCAAACGTAAAACGGCTTGTCCCGCGTCATCGGCGGGGGTCATAACGTGACTCCCTTA
ATTCTCCGCTCATGATCTTGATCCCCTGCGCCATCAGATCCTTGGCGGCAAGAAAGCCATCCAGTTTACTTTGCAGGGCTTCCCAACCTTACCAGAGGGCGCCCCAGCTGG
CAATTCCGGTTCGCTTGCTGTCCATAAAACCGCCCAGTCTAGCTATCGCCATGTAAGCCCACTGCAAGCTACCTGCTTTCTCTTTGCGCTTGCGTTTTCCCTTGTCCAGATA
GCCCAGTAGCTGACATTCATCCGGGGTCAGCACCGTTTCTGCGGACTGGCTTTCTACGTGTTCCGCTTCCTTTAGCAGCCCTTGCGCCCTGAGTGCTTGCGGCAGCGTGAA
GCTGGCGCGCCGCTCTAGCAGAAGGCATGTTGTTGTGACTCCGAGGGGTTGCCTCAAACTCTATCTTATAACCGGCGTGGAGGCATGGAGGCAAGGGCATTTTGGTAATT
TAAGTAGTTAGTGGAAAATGACGTCATTTACTTAAAGACGAAGTCTTGCGACAAGGGGGGCCCACGCCGAATTTTAATATTACCGGCGTGGCCCCACCTTATCGCGAGTG
CTTTAGCACGAGCGGTCCAGATTTAAAGTAGAAAAGTTCCCGCCCACTAGGGTTAAAGGTGTTCACACTATAAAAGCATATACGATGTGATGGTATTTGATGGAGCGTAT
ATTGTATCAGGTATTTCCGTCGGATACGAATTATTCGTACGACCCTCCTGCAGGCATTCGGAGTTTTTGTATCTTGTTTCATAGTTTGTCCCAGGATTAGAATGATTAGGCA
TCGAACCTTCAAGAATTTGATTGAATAAAACATCTTCATTCTTAAGATATGAAGATAATCTTCAAAAGGCCCCTGGGAATCTGAAAGAAGAGAAGCAGACCCATTTATAT
GGGAAAGAACAATAGTATTTCTTATATAGGCCCATTTAAGTTGAAAACAATCTTCAAAAGTCCCACATCGCTTAGATAAGAAAACGAAGCTGAGTTTATATACAGCTAG
AGTCGAAGTAGTGATTGAGACCTCTAGAGAGCTCGGTCTCCTTTTTTTGTTTTTTGAAGAGCTTTGCTCTTCTTCCGAGTGTACTTCAAGTCAGTTGGAAATCAAT
AAAATGATTATTTTATGAATATATTTCATTGTGCAAGTAGATAGAAATTACATATGTTACATAACACACGAAATAAACAAAAAAACACAATCCAAAACAAACACCCCAA
ACAAAATAACACTATATATATCCTCGTATGAGGAGAGGCACGTTCAGTGACTCGACGATTCCCGAGCAAAAAAAGTCTCCCCGTCACACATATAGTGGGTGACGCAATT
ATCTTCAAAGTAATCCTTCTGTTGACTTGTCATTGATAACATCCAGTCTTCGTCAGGATTGCAAAGAATTATAGAAGGGATCCCACCTTTTATTTCTTCTTTTTCCATATT
TAGGGTTGACAGTGAAATCAGACTGGCAACCTATTAATTGCTTCCACAATGGGACGAACTTGAAGGGGATGTCGTCGATGATATTATAGGTGGCGTGTTCATCGTAGTTG
GTGAAGTCGATGGTCCCGTTCCAGTAGTTGTGTCGCCCGAGACTTCTAGCCCAGGTGGTCTTTCCGGTACGAGTTGGTCCGCAGATGTAGAGGCTGGGGTGTCTGACCCC
AGTCCTTCCCTCATCCTGGTTAGATCGGCCATCCACTCAAGGTCAGATTGTGCTTGATCGTAGGAGACAGGATGTATGAAAGTGTAGGCATCGATGCTTACATGATATAG
GTGCGTCTCTCTCCAGTTGTGCAGATCTTCGTGGCAGCGGAGATCTGATTCTGTGAAGGGCGACACGTACTGCTCAGGTTGTGGAGGAAATAATTTGTTGGCTGAATATT
CCAGCCATTGAAGCTTTGTTGCCCATTCATGAGGGAACTCTTCTTTGATCATGTCAAGATACTCCTCCTTAGACGTTGCAGTCTGGATAATAGTTCGCCATCGTGCGTCAG
ATTTGCGAGGAGACACCTTATGATCTCGGAAATCTCCTCTGGTTTTAATATCTCCGTCCTTTGATATGTAATCAAGGACTTGTTTAGAGTTTCTAGCTGGCTGGATATTAGG
GTGATTTCCTTCAAAATCGAAAAAAGAAGGATCCCTAATACAAGGTTTTTTATCAAGCTGGATAAGAGCATGATAGTGGGTAGTGCCATCTTGATGAAGCTCAGAAGCA
ACACCAAGGAAGAAAATAAGAAAAGGTGTGAGTTTCTCCCAGAGAAACTGGAATAAATCATCTCTTTGAGATGAGCACTTGGGGTAGGTAAGGAAAACATATTTAGATT
GGAGTCTGAAGTTCTTGCTAGCAGAAGGCATGTTGTTGTGACTCCGAGGGGTTGCCTCAAACTCTATCTTATAACCGGCGTGGAGGCATGGAGGCAAGGGCATTTTGGTA
ATTTAAGTAGTTAGTGGAAAATGACGTCATTTACTTAAAGACGAAGTCTTGCGACAAGGGGGGCCCACGCCGAATTTTAATATTACCGGCGTGGCCCCACCTTATCGCGA
GTGCTTTAGCACGAGCGGTCCAGATTTAAAGTAGAAAAGTTCCCGCCCACTAGGGTTAAAGGTGTTCACACTATAAAAGCATATACGATGTGATGGTATTTGATGGAGCG
TATATTGTATCAGGTATTTCCGTCGGATACGAATTATTCGTACGGCCGGACCGGTCCCCTAGGCCGGCCAATTCGAGATCGGCCGCGGCTGAGTGGCTCCTTCAATCGTTG
CGGTTCTGTCAGTTCCAAACGTAAAACGGCTTGTCCCGCGTCATCGGCGGGGGTCATAACGTGACTCCCTTAATTCTCCGCTCATGATCAGATTGTCGTTTCCCGCCTTCA
GTTTAAACTATCAGTGTTTGACAGGATATATTGGCGGGTAAACCTAAGAGAAAAGAGCGTTTATTAGAATAATCGGATATTTAAAAGGGCGTGAAAAGGTTTATCCGTTC
GTCCATTTGTATGTGCATGCCAACCACAGGGTTCCCCAGATCTGGCGCCGGCCAGCGAGACGAGCAAGATTGGCCGCCGCCCGAAACGATCCGACAGCGCGCCCAGCAC
AGGTGCGCAGGCAAATTGCACCAACGCATACAGCGCCAGCAGAATGCCATAGTGGGCGGTGACGTCGTTCGAGTGAACCAGATCGCGCAGGAGGCCCGGCAGCACCGG
CATAATCAGGCCGATGCCGACAGCGTCGAGCGCGACAGTGCTCAGAATTACGATCAGGGGTATGTTGGGTTTCACGTCTGGCCTCCGGAGACTGTCATACGCGTAAAAA
GGCCGCGTTGCTGGCGTTTTTCCATAGGCTCCGCCCCCCTGACGAGCATCACAAAAATCGACGCTCAAGTCAGAGGTGGCGAAACCCGACAGGACTATAAAGATACCAG
GCGTTTCCCCCTGGAAGCTCCCTCGTGCGCTCTCCTGTTCCGACCCTGCCGCTTACCGGATACCTGTCCGCCTTTCTCCCTTCGGGAAGCGTGGCGCTTTCTCATAGCTCAC
GCTGTAGGTATCTCAGTTCGGTGTAGGTCGTTCGCTCCAAGCTGGGCTGTGTGCACGAACCCCCCGTTCAGCCCGACCGCTGCGCCTTATCCGGTAACTATCGTCTTGAGT
CCAACCCGGTAAGACACGACTTATCGCCACTGGCAGCAGCCACTGGTAACAGGATTAGCAGAGCGAGGTATGTAGGCGGTGCTACAGAGTTCTTGAAGTGGTGGCCTAA
CTACGGCTACACTAGAAGGACAGTATTTGGTATCTGCGCTCTGCTGAAGCCAGTTACCTTCGGAAAAAGAGTTGGTAGCTCTTGATCCGGCAAACAAACCACCGCTGGTA
GCGGTGGTTTTTTTGTTTGCAAGCAGCAGATTACGCGCAGAAAAAAAGGATCTCAAGAAGATCCTTTGATCTTTTCTACGGGGTCTGACGCTCAGTGGAACGAAAACTCA
CGTTAAGGGATTTTGGTCATGAGATTATCAAAAAGGATCTTCACCTAGATCCTTTTAAATTAAAAATGAAGTTTTAAATCAATCTAAAGTATATATGAGTAAACTTGGTCT
GCAGTTGCCATGTTTTACGGCAGTGAGAGCAGAGATAGCGCTGATGTCCGGCGGTGCTTTTGCCGTTACGCACCACCCCGTCAGTAGCTGAACAGGAGGGACAGCTGAT
AGACACAGAAGCCACTGGAGCACCTCAAAAACACCATCATACACTAAATCAGTAAGTTGGCAGCATCACCCATAATTGTGGTTTCAAAATCGGCTCCGTCGATACTATGT
TATACGCCAACTTTGAAAACAACTTTGAAAAAGCTGTTTTCTGGTATTTAAGGTTTTAGAATGCAAGGAACAGTGAATTGGAGTTCGTCTTGTTATAATTAGCTTCTTGGG
GTATCTTTAAATACTGTAGAAAAGAGGAAGGAAATAATAAATGGCTAAAATGAGAATATCACCGGAATTGAAAAAACTGATCGAAAAATACCGCTGCGTAAAAGATAC
GGAAGGAATGTCTCCTGCTAAGGTATATAAGCTGGTGGGAGAAAATGAAAACCTATATTTAAAAATGACGGACAGCCGGTATAAAGGGACCACCTATGATGTGGAACG
GGAAAAGGACATGATGCTATGGCTGGAAGGAAAGCTGCCTGTTCCAAAGGTCCTGCACTTTGAACGGCATGATGGCTGGAGCAATCTGCTCATGAGTGAGGCCGATGGC
GTCCTTTGCTCGGAAGAGTATGAAGATGAACAAAGCCCTGAAAAGATTATCGAGCTGTATGCGGAGTGCATCAGGCTCTTTCACTCCATCGACATATCGGATTGTCCCTA
TACGAATAGCTTAGACAGCCGCTTAGCCGAATTGGATTACTTACTGAATAACGATCTGGCCGATGTGGATTGCGAAAACTGGGAAGAAGACACTCCATTTAAAGATCCG
CGCGAGCTGTATGATTTTTTAAAGACGGAAAAGCCCGAAGAGGAACTTGTCTTTTCCCACGGCGACCTGGGAGACAGCAACATCTTTGTGAAAGATGGCAAAGTAAGTG
GCTTTATTGATCTTGGGAGAAGCGGCAGGGCGGACAAGTGGTATGACATTGCCTTCTGCGTCCGGTCGATCAGGGAGGATATCGGGGAAGAACAGTATGTCGAGCTATT
TTTTGACTTACTGGGGATCAAGCCTGATTGGGAGAAAATAAAATATTATATTTTACTGGATGAATTGTTTTAGTACCTAGATGTGGCGCAACGATGCCGGCGACAAGCAG
GAGCGCACCGACTTCTTCCGCATCAAGTGTTTTGGCTCTCAGGCCGAGGCCCACGGCAAGTATTTGGGCAAGGGGTCGCTGGTATTCGTGCAGGGCAAGATTCGGAATAC
CAAGTACGAGAAGGACGGCCAGACGGTCTACGGGACCGACTTCATTGCCGATAAGGTGGATTATCTGGACACCAAGGCACCAGGCGGGTCAAATCAGGAATAAGGGCA
CATTGCCCCGGCGTGAGTCGGGGCAATCCCGCAAGGAGGGTGAATGAATCGGACGTTTGACCGGAAGGCATACAGGCAAGAACTGATCGACGCGGGGTTTTCCGCCGA
GGATGCCGAAACCATCGCAAGCCGCACCGTCATGCGTGCGCCCCGCGAAACCTTCCAGTCCGTCGGCTCGATGGTCCAGCAAGCTACGGCCAAGATCGAGCGCGACAGC
GTGCAACTGGCTCCCCCTGCCCTGCCCGCGCCATCGGCCGCCGTGGAGCGTTCGCGTCGTCTCGAACAGGAGGCGGCAGGTTTGGCGAAGTCGATGACCATCGACACGC
GAGGAACTATGACGACCAAGAAGCGAAAAACCGCCGGCGAGGACCTGGCAAAACAGGTCAGCGAGGCCAAGCAGGCCGCGTTGCTGAAACACACGAAGCAGCAGATC
AAGGAAATGCAGCTTTCCTTGTTCGATATTGCGCCGTGGCCGGACACGATGCGAGCGATGCCAAACGACACGGCCCGCTCTGCCCTGTTCACCACGCGCAACAAGAAAA
TCCCGCGCGAGGCGCTGCAAAACAAGGTCATTTTCCACGTCAACAAGGACGTGAAGATCACCTACACCGGCGTCGAGCTGCGGGCCGACGATGACGAACTGGTGTGGCA
GCAGGTGTTGGAGTACGCGAAGCGCACCCCTATCGGCGAGCCGATCACCTTCACGTTCTACGAGCTTTGCCAGGACCTGGGCTGGTCGATCAATGGCCGGTATTACACGA
AGGCCGAGGAATGCCTGTCGCGCCTACAGGCGACGGCGATGGGCTTCACGTCCGACCGCGTTGGGCACCTGGAATCGGTGTCGCTGCTGCACCGCTTCCGCGTCCTGGAC

CGTGGCAAGAAAACGTCCCGTTGCCAGGTCCTGATCGACGAGGAAATCGTCGTGCTGTTTGCTGGCGACCACTACACGAAATTCATATGGGAGAAGTACCGCAAGCTGT
CGCCGACGGCCCGACGGATGTTCGACTATTTCAGCTCGCACCGGGAGCCGTACCCGCTCAAGCTGGAAACCTTCCGCCTCATGTGCGGATCGGATTCCACCCGCGTGAAG
AAGTGGCGCGAGCAGGTCGGCGAAGCCTGCGAAGAGTTGCGAGGCAGCGGCCTGGTGGAACACGCCTGGGTCAATGATGACCTGGTGCATTGCAAACGCTAGGGCCTT
GTGGGGTCAGTTCCGGCTGGGGGTTCAGCAGCCAGCGCTTTACTGGCATTTCAGGAACAAGCGGGCACTGCTCGACGCACTTGCTTCGCTCAGTATCGCTCGGGACGCAC
GGCGCGCTCTACGAACTGCCGATAAACAGAGGATTAAAATTGACAATTCAATGGCAAGGACTGCCAGCGCTGCCATTTTTGGGGTGAGGCCGTTCGCGGCCGAGGGGCG
CAGCCCCTGGGGGGATGGGAGGCCCGCGTTAGCGGGCCGGGAGGGTTCGAGAAGGGGGGGCACCCCCCTTCGGCGTGCGCGGTCACGCGCACAGGGCGCAGCCCTGGT
TAAAAACAAGGTTTATAAATATTGGTTTAAAAGCAGGTTAAAAGACAGGTTAGCGGTGGCCGAAAAACGGGCGGAAACCCTTGCAAATGCTGGATTTTCTGCCTGTGGA
CAGCCCCTCAAATGTCAATAGGTGCGCCCCTCATCTGTCAGCACTCTGCCCCTCAAGTGTCAAGGATCGCGCCCCTCATCTGTCAGTAGTCGCGCCCCTCAAGTGTCAATA
CCGCAGGGCACTTATCCCCAGGCTTGTCCACATCATCTGTGGGAAACTCGCGTAAAATCAGGCGTTTTCGCCGATTTGCGAGGCTGGCCAGCTCCACGTCGCCGGCCGAA
ATCGAGCCTGCCCCTCATCTGTCAACGCCGCGCCGGGTGAGTCGGCCCCTCAAGTGTCAACGTCCGCCCCTCATCTGTCAGTGAGGGCCAAGTTTTCCGCGAGGTATCCA
CAACGCCGGCGGCCGCGGTGTCTCGCACACGGCTTCGACGGCGTTTCTGGCGCGTTTGCAGGGCCATAGACGGCCGCCAGCCCAGCGGCGAGGGCAACCAGCCCGGTGA
GCGTCGCAAAGGCGCTCGGTCTTGCCTTGCTCGTCGAGATCTGGGGTCGATCAGCCGGGGATGCATCAGGCCGACAGTCGGAACTTCGGGTCCCCGACCTGTACCATTCG
GTGAGCAATGGATAGGGGAGTTGATATCGTCAACGTTCACTTCTAAAGAAATAGCGCCACTCAGCTTCCTCAGCGGCTTTATCCAGCGATTTCCTATTATGTCGGCATAG
TTCTCAAGATCGACAGCCTGTCACGGTTAAGCGAGAAATGAATAAGAAGGCTGATAATTCGGATCTCTGCGAGGGAGATGATATTTGATCACAGGCAGCAACGCTCTGT
CATCGTTACAATCAACATGCTACCCTCCGCGAGATCATCCGTGTTTCAAACCCGGCAGCTTAGTTGCCGTTCTTCCGAATAGCATCGGTAACATGAGCAAAGTCTGCCGC
CTTACAACGGCTCTCCCGCTGACGCCGTCCCGGACTGATGGGCTGCCTGTATCGAGTGGTGATTTTGTGCCGAGCTGCCGGTCGGGGAGCTGTTGGCTGGCTGGTGGCAG
GATATATTGTGGTGTAAACAAATTGACGCTTAGACAACTTAATAACACATTGCGGACGTTTTTAATGTACTGGGGTGGTTTTTCTTTTCACCAGTGAGACGGGCAACAGCT
GATTGCCCTTCACCGCCTGGCCCTGAGAGAGTTGCAGCAAGCGGTCCACGCTGGTTTGCCCCAGCAGGCGAAAATCCTGTTTGATGGTGGTTCCGAAATCGGCAAAATCC
CTTATAAATCAAAAGAATAGCCCGAGATAGGGTTGAGTGTTGTTCCAGTTTGGAACAAGAGTCCACTATTAAAGAACGTGGACTCCAACGTCAAAGGGCGAAAAACCGT
CTATCAGGGCGATGGCCCACTACGTGAACCATCACCCAAATCAAGTTTTTTGGGGTCGAGGTGCCGTAAAGCACTAAATCGGAACCCTAAAGGGAGCCCCCGATTTAGA
GCTTGACGGGGAAAGCCGGCGAACGTGGCGAGAAAGGAAGGGAAGAAAGCGAAAGGAGCGGGCGCCATTCAGGCTGCGCAACTGTTGGGAAGGG

**SpCas12f gRNA:** SpCas12f1 gRNA scaffold (Bigelyte et al. 2021) with restriction enzyme sites for cloning the guide sequence as oligonucleotides. The SpCas12f1 gRNA scaffold is marked in yellow. The BsaI restriction enzyme site for cloning annealed oligonucleotides is underlined.

GATTTACTCTGTTTCGCGCGCCAGGGCAGTTAGGTGCCCTAAAAGAGCGAAGTGGCCGAAAGGAAAGGCTAACGCTTCTCTAACGCTACGGCGACCTTGGCGAAATGCC
ATCAATACCACGCGGCCCGAAAGGGTTCGCGCGAAACTGAGTAATGAAAGTCGCATCTTGCGTAAGCGCGTGGATTGAAACGGAGACCTCTAGAGAGCTCGGTCTCC

**WugRNAv2:** Improved AsCas12f1 gRNA scaffold (Wu et al. 2021), referred to as WugRNAv2, with restriction enzyme sites for cloning the guide sequence as oligonucleotides. The WugRNAv2 sequence is marked in yellow. The BsaI restriction enzyme site for cloning annealed oligonucleotides is underlined.

GATTCGTCGGTTCAGCGACGATAAGCCGAGAAGTGCCAATAAAAGTGGTTTGGTAACGCTCGGTAAGGTAGCCAAAAGGCTGAAACTCCGTGCACAAAGACCGCACGG
ACGCTTCACATATAGCTTGTGGAGTGTGAACGGAGACCTCTAGAGAGCTCGGTCTCC

**KimgRNAv4.1:** Improved Un1Cas12f1 gRNA scaffold (Kim et al. 2022), referred to as KimgRNAv4.1, with restriction enzyme sites for cloning the guide sequence as oligonucleotides. The KimgRNAv4.1 sequence is marked in yellow. The BsaI restriction enzyme site for cloning annealed oligonucleotides is underlined.

GACCGCTTCACTTAGAGTGAAGGTGGGCTGCTTGCATCAGCCTAATGTCGAGAAGTGCTTTCTTCGGAAAGTAACCCTCGAAACAAAGAAAGGAATGCAACGGAGACCT
CTAGAGAGCTCGGTCTCC

**Cas12j-2 gRNA:** Processed 25 nt Cas12j-2 gRNA scaffold (Pausch et al. 2020) with restriction enzyme sites for cloning the guide sequence as oligonucleotides. The Cas12j-2 gRNA scaffold is marked in yellow. The BsaI restriction enzyme site for cloning annealed oligonucleotides is underlined.

GCAACGATTGCCCCTCACGAGGGGACGGAGACCTTTGGTCTCC

**Cas9 sgRNA scaffold:** SpCas9 sgRNA scaffold with restriction enzyme sites for cloning the guide sequence as oligonucleotides. The KimgRNAv4.1 sequence is marked in yellow. The BsaI restriction enzyme site for cloning annealed oligonucleotides is underlined.

GAGACCGGCCACGGGTCTCGGTTTTAGAGCTAGAAATAGCAAGTTAAAATAAGGCTAGTCCGTTATCAACTTGAAAAAGTGGCACCGAGTCGGTGC

**Table S2. Guide sequence oligonucleotides used in this study.**

The below oligonucleotides were annealed and cloned into GVR-gRNA vectors to form guide sequences. The guide sequences are highlighted in yellow in the forward oligo sequence. F indicates the forward oligonucleotide and R indicates the reverse oligonucleotide.

| Oligo name | gRNA # | Oligo sequence (5’ - 3’) |
|---|---|---|
| Cas9PDSg1-F | SpCas9 PDSgRNA1 | TGATTGTTGGTAGTAGCGACTCCATG |
| Cas9PDSg1-R | SpCas9 PDSgRNA1 | AAACCATGGAGTCGCTACTACCAACA |
| Cas9PDSg4-F | SpCas9 PDSgRNA4 | TGATTGGATGGAGATTGGTACGAGAC |
| Cas9PDSg4-R | SpCas9 PDSgRNA4 | AAACGTCTCGTACCAATCTCCATCCA |
| SpCas12fPDSg1-F | SpCas12f1 PDSgRNA1 | AAACCTGAAGCTCTTCCTGCGC |
| SpCas12fPDSg1-R | SpCas12f1 PDSgRNA1 | AAAAGCGCAGGAAGAGCTTCAG |
| SpCas12fPDSg2-F | SpCas12f1 PDSgRNA2 | AAACAACATAAGATTGCCCTCC |
| SpCas12fPDSg2-R | SpCas12f1 PDSgRNA2 | AAAAGGAGGGCAATCTTATGTT |
| SpCas12fPDSg3-F | SpCas12f1 PDSgRNA3 | AAACATTGCCATGTCAAAGGCA |
| SpCas12fPDSg3-R | SpCas12f1 PDSgRNA3 | AAAATGCCTTTGACATGGCAAT |
| SpCas12fPDSg6-F | SpCas12f1 PDSgRNA6 | AAACCTGTGATAAATGTCCATA |
| SpCas12fPDSg6-R | SpCas12f1 PDSgRNA6 | AAAATATGGACATTTATCACAG |
| SpCas12fPDSg7-F | SpCas12f1 PDSgRNA7 | AAACAGCCGCTTTGATTTTCCT |
| SpCas12fPDSg7-R | SpCas12f1 PDSgRNA7 | AAAAAGGAAAATCAAAGCGGCT |
| SpCas12fPDSg8-F | SpCas12f1 PDSgRNA8 | AAACGTGAGTTTAGTCTGACTT |

| | | |
|---|---|---|
| SpCas12fPDSg8-R | SpCas12f1 PDSgRNA8 | AAAAAAGTCAGACTAAACTCAC |
| SpCas12fPDSg9-F | SpCas12f1 PDSgRNA9 | AAACGGCAGATCAGAGCAAAGC |
| SpCas12fPDSg9-R | SpCas12f1 PDSgRNA9 | AAAAGCTTTGCTCTGATCTGCC |
| SpCas12fPDSg10-F | SpCas12f1 PDSgRNA10 | AAACAATGGAAGGTGCTGTCTT |
| SpCas12fPDSg10-R | SpCas12f1 PDSgRNA10 | AAAAAAGACAGCACCTTCCATT |
| SpCas12fPDSg3-20nt-F | SpCas12f1 PDSgRNA3-20nt | AAACATTGCCATGTCAAAGGCACT |
| SpCas12fPDSg3-20nt-R | SpCas12f1 PDSgRNA3-20nt | AAAAAGTGCCTTTGACATGGCAAT |
| SpCas12fPDSg6-20nt-F | SpCas12f1 PDSgRNA6-20nt | AAACCTGTGATAAATGTCCATATA |
| SpCas12fPDSg6-20nt-R | SpCas12f1 PDSgRNA6-20nt | AAAATATATGGACATTTATCACAG |
| CasMINIPDSg2-F | CasMINIv3.1 PDSgRNA2 | CAACGTAGTAGCGACTCCATGGGG |
| CasMINIPDSg2-R | CasMINIv3.1 PDSgRNA2 | AAAACCCCATGGAGTCGCTACTAC |
| CasMINIPDSg3-F | CasMINIv3.1 PDSgRNA3 | CAACTGACCAGCATCTGCCAGATA |
| CasMINIPDSg3-R | CasMINIv3.1 PDSgRNA3 | AAAATATCTGGCAGATGCTGGTCA |
| CasMINIPDSg4-F | CasMINIv3.1 PDSgRNA4 | CAACATGGCGCAGGAAGAGCTTCA |
| CasMINIPDSg4-R | CasMINIv3.1 PDSgRNA4 | AAAATGAAGCTCTTCCTGCGCCAT |
| CasMINIPDSg5-F | CasMINIv3.1 PDSgRNA5 | CAACCTATTGGACTCTTGCCAGCA |
| CasMINIPDSg5-R | CasMINIv3.1 PDSgRNA5 | AAAATGCTGGCAAGAGTCCAATAG |
| CasMINIPDSg6-F | CasMINIv3.1 PDSgRNA6 | CAACTGAAGTTAAGTGCCTTTGAC |
| CasMINIPDSg6-R | CasMINIv3.1 PDSgRNA6 | AAAAGTCAAAGGCACTTAACTTCA |
| CasMINIPDSg7-F | CasMINIv3.1 PDSgRNA7 | CAACTACTGAATAATGGCAGTACA |
| CasMINIPDSg7-R | CasMINIv3.1 PDSgRNA7 | AAAATGTACTGCCATTATTCAGTA |
| CasMINIPDSg8-F | CasMINIv3.1 PDSgRNA8 | CAACATTGTACTGCCATTATTCAG |
| CasMINIPDSg8-R | CasMINIv3.1 PDSgRNA8 | AAAACTGAATAATGGCAGTACAAT |
| CasMINIPDSg10-F | CasMINIv3.1 PDSgRNA10 | CAACATTGCTTTGAACAGATTTCT |
| CasMINIPDSg10-R | CasMINIv3.1 PDSgRNA10 | AAAAAGAAATCTGTTCAAAGCAAT |
| AsCas12fPDSg1-F* | AsCas12f PDSgRNA1 | GAACGTAGTAGCGACTCCATGGGG |
| AsCas12fPDSg3-F* | AsCas12f PDSgRNA3 | GAACATGGCGCAGGAAGAGCTTCA |
| AsCas12fPDSg4-F* | AsCas12f PDSgRNA4 | GAACCTATTGGACTCTTGCCAGCA |
| AsCas12fPDSg6-F* | AsCas12f PDSgRNA6 | GAACTACTGAATAATGGCAGTACA |
| AsCas12fPDSg11-F | AsCas12f PDSgRNA11 | GAACGAGGGCAATCTTATGTTGAA |
| AsCas12fPDSg11-R | AsCas12f PDSgRNA11 | AAAATTCAACATAAGATTGCCCTC |
| AsCas12fPDSg12-F | AsCas12f PDSgRNA12 | GAACAGCTTCAACATAAGATTGCC |
| AsCas12fPDSg12-R | AsCas12f PDSgRNA12 | AAAAGGCAATCTTATGTTGAAGCT |
| AsCas12fPDSg14-F | AsCas12f PDSgRNA14 | GAACCCCAAATATGCAGAACCTGT |
| AsCas12fPDSg14-R | AsCas12f PDSgRNA14 | AAAAACAGGTTCTGCATATTTGGG |
| AsCas12fPDSg15-F | AsCas12f PDSgRNA15 | GAACCAGAATATGTGCAACCCAGT |
| AsCas12fPDSg15-R | AsCas12f PDSgRNA15 | AAAAACTGGGTTGCACATATTCTG |
| CasPhiPDSg3-F | Cas12j-2 PDSgRNA3 | GGACGATAATCAATGCAGACTACC |
| CasPhiPDSg3-R | Cas12j-2 PDSgRNA3 | AAAAGGTAGTCTGCATTGATTATC |
| CasPhiPDSg4-F | Cas12j-2 PDSgRNA4 | GGACACTATTTGGAGGCGGCGTTA |
| CasPhiPDSg4-R | Cas12j-2 PDSgRNA4 | AAAATAACGCCGCCTCCAAATAGT |
| CasPhiPDSg5-F | Cas12j-2 PDSgRNA5 | GGACCCTCCAGCAATATCGGTTTG |
| CasPhiPDSg5-R | Cas12j-2 PDSgRNA5 | AAAACAAACCGATATTGCTGGAGG |
| CasPhiPDSg6-F | Cas12j-2 PDSgRNA6 | GGACGTACGAGACTGGGTTGCACA |
| CasPhiPDSg6-R | Cas12j-2 PDSgRNA6 | AAAATGTGCAACCCAGTCTCGTAC |
| CasPhiPDSg8-F | Cas12j-2 PDSgRNA8 | GGACACACTTAAACCGTCTTGAGC |
| CasPhiPDSg8-R | Cas12j-2 PDSgRNA8 | AAAAGCTCAAGACGGTTTAAGTGT |
| CasPhiPDSg11-F | Cas12j-2 PDSgRNA11 | GGACCTGTGATAAATGTCCATATA |
| CasPhiPDSg11-R | Cas12j-2 PDSgRNA11 | AAAATATATGGACATTTATCACAG |
| CasPhiPDSg12-F | Cas12j-2 PDSgRNA12 | GGACAATGGAAGGTGCTGTCTTAT |
| CasPhiPDSg12-R | Cas12j-2 PDSgRNA12 | AAAAATAAGACAGCACCTTCCATT |
| CasPhiPDSg13-F | Cas12j-2 PDSgRNA13 | GGACTTCAGTATAAAACATTTGAC |

| | | |
|---|---|---|
| CasPhiPDSg13-R | Cas12j-2 PDSgRNA13 | AAAAGTCAAATGTTTTATACTGAA |

*Note: AsCas12fPDSg1-F, AsCas12fPDSg3-F, AsCas12fPDSg4-F, AsCas12fPDSg6-F had the same guide sequence and were annealed with CasMINIPDSg2-R, CasMINIPDSg4-R, CasMINIPDSg5-R, CasMINIPDSg7-R, respectively.

**Table S3. PCR primers used in this study for targeted amplicon sequencing.**

Nextera-tagged PCR primer combinations used for amplification of the target region and amplicon sequencing at the Australian Genome Research Facility. The Nextera tag sequence for the first primer combination is highlighted in yellow followed by the gene-specific sequence.

| Primer Code | Primer sequence (5’ - 3’) | gRNA # |
|---|---|---|
| P#250 | TCGTCGGCAGCGTCAGATGTGTATAAGAGACAGGAGAGTCCAAGGT AATTCAGCT | CasMINIv3.1 PDSgRNA2, SpCas9 PDSgRNA1, AsCas12f1 PDSgRNA1, |
| P#251 | GTCTCGTGGGCTCGGAGATGTGTATAAGAGACAGCAGCATCACACT TTCGCATTCAA | |
| P#277 | TCGTCGGCAGCGTCAGATGTGTATAAGAGACAGGAAGCTGTAGCAT GATTCTAATT | Cas12j-2 PDSgRNA5, CasMINIv3.1 PDSgRNA3 |
| P#278 | GTCTCGTGGGCTCGGAGATGTGTATAAGAGACAGCAAACAGAAGCC TTCCTGA | |
| P#254 | TCGTCGGCAGCGTCAGATGTGTATAAGAGACAGCTATCTATGCGTT ATTACTTCTACAGG | CasMINIv3.1 PDSgRNA4, SpCas12f1 PDSgRNA1, SpCas12f1 PDSgRNA7, AsCas12f1 PDSgRNA3 |
| P#283 | GTCTCGTGGGCTCGGAGATGTGTATAAGAGACAGCTAATCAATTTC ATACCTTATTGG | |
| P#284 | TCGTCGGCAGCGTCAGATGTGTATAAGAGACAGATGCGTTATTACTT CTACAGGAAT | CasMINIv3.1 PDSgRNA5, Cas12j-2 PDSgRNA8, SpCas12f1 PDSgRNA2, AsCas12f1 PDSgRNA4, AsCas12f1 PDSgRNA11 AsCas12f1 PDSgRNA12 |
| P#285 | GTCTCGTGGGCTCGGAGATGTGTATAAGAGACAGATGCAAGTCAAC TATGTCCTCAAGG | |
| P#286 | TCGTCGGCAGCGTCAGATGTGTATAAGAGACAGAGCAGCAGCTATT TGCTTAA | CasMINIv3.1 PDSgRNA6, CasMINIv3.1 PDSgRNA10, SpCas12f1 PDSgRNA3 |
| P#287 | GTCTCGTGGGCTCGGAGATGTGTATAAGAGACAGTATGTCAGGTCC CTAACATGA | |
| P#336 | TCGTCGGCAGCGTCAGATGTGTATAAGAGACAGTGCAGGAGAAACA TGGTTCA | CasMINIv3.1 PDSgRNA7, CasMINIv3.1 PDSgRNA8, Cas12j-2 PDSgRNA13, SpCas12f1 PDSgRNA8, AsCas12f1 PDSgRNA6 |
| P#337 | GTCTCGTGGGCTCGGAGATGTGTATAAGAGACAGATGGATATTATA CCTGGAGTGGC | |
| P#364 | TCGTCGGCAGCGTCAGATGTGTATAAGAGACAGTGAATGCGAAAGT GTGATGCTG | Cas12j-2 PDSgRNA3, Cas12j-2 PDSgRNA4 |
| P#13-G | GTCTCGTGGGCTCGGAGATGTGTATAAGAGACAGTAATAGAATGAT CTTCCTTCCAAAG | |
| P#279 | TCGTCGGCAGCGTCAGATGTGTATAAGAGACAGTTGAATTAGCATT AATCTTTATGTG | Cas12j-2 PDSgRNA6, SpCas9 PDSgRNA4, AsCas12f1 PDSgRNA15 |
| P#280 | GTCTCGTGGGCTCGGAGATGTGTATAAGAGACAGTCGACTGACAAA AATTGGCTAT | |
| P#290 | TCGTCGGCAGCGTCAGATGTGTATAAGAGACAGGCCTGAAGACTGG AAAGAG | Cas12j-2 PDSgRNA11, SpCas12f1 PDSgRNA6 |
| P#291 | GTCTCGTGGGCTCGGAGATGTGTATAAGAGACAGGGAGGACCACAT AAACATGT | |
| P#292 | TCGTCGGCAGCGTCAGATGTGTATAAGAGACAGGTGCCAGGTTGTG AACCCTG | Cas12j-2 PDSgRNA12, SpCas12f1 PDSgRNA10 |
| P#293 | GTCTCGTGGGCTCGGAGATGTGTATAAGAGACAGCTCTACCGCAGC AAAGATGACAA | |
| P#281 | TCGTCGGCAGCGTCAGATGTGTATAAGAGACAGGTCAGTCGATCTG TATTCTGCC | AsCas12f1 PDSgRNA14 |
| P#282 | GTCTCGTGGGCTCGGAGATGTGTATAAGAGACAGGTTAGGCATCGC AAATATCA | |
| P#294 | TCGTCGGCAGCGTCAGATGTGTATAAGAGACAGAGAGTGGATAAAT CGTAGTGACTCA | SpCas12f1 PDSgRNA9 |
| P#295 | GTCTCGTGGGCTCGGAGATGTGTATAAGAGACAGAGGAATAGATTC CACGTGGAC | |

## References

Bigelyte G, Young JK, Karvelis T, Budre K, Zedaveinyte R, Djukanovic V, Van Ginkel E, Paulraj S, Gasior S, Jones S, Feigenbutz L, Clair GS, Barone P, Bohn J, Acharya A, Zastrow-Hayes G, Henkel-Heinecke S, Silanskas A, Seidel R, Siksnys V (2021) Miniature type V-F CRISPR-Cas nucleases enable targeted DNA modification in cells. Nature Communications 12:6191

Bombarely A, Rosli HG, Vrebalov J, Moffett P, Mueller LA, Martin GB (2012) A Draft Genome Sequence of Nicotiana benthamiana to Enhance Molecular Plant-Microbe Biology Research. Molecular Plant-Microbe Interactions® 25: 1523-1530

Clement K, Rees H, Canver MC, Gehrke JM, Farouni R, Hsu JY, Cole MA, Liu DR, Joung JK, Bauer DE, Pinello L (2019) CRISPResso2 provides accurate and rapid genome editing sequence analysis. Nature Biotechnology 37: 224-226

Diamos AG, Rosenthal SH, Mason HS (2016) 5' and 3' Untranslated Regions Strongly Enhance Performance of Geminiviral Replicons in Nicotiana benthamiana Leaves. **Front Plant Sci** 7: 200

Edwards K, Johnstone C, Thompson C (1991) A simple and rapid method for the preparation of plant genomic DNA for PCR analysis. Nucleic Acids Research 19: 1349

Kim DY, Lee JM, Moon SB, Chin HJ, Park S, Lim Y, Kim D, Koo T, Ko JH, Kim YS (2022) Efficient CRISPR editing with a hypercompact Cas12f1 and engineered guide RNAs delivered by adeno-associated virus. Nature Biotechnology 40: 94-102

Pausch P, Al-Shayeb B, Bisom-Rapp E, Tsuchida CA, Li Z, Cress BF, Knott GJ, Jacobsen SE, Banfield JF, Doudna JA (2020) CRISPR-CasΦ from huge phages is a hypercompact genome editor. Science 369: 333-337

Wu Z, Zhang Y, Yu H, Pan D, Wang Y, Wang Y, Li F, Liu C, Nan H, Chen W, Ji Q (2021) Programmed genome editing by a miniature CRISPR-Cas12f nuclease. Nature Chemical Biology 17: 1132-1138

Xie K, Minkenberg B, Yang Y (2015) Boosting CRISPR/Cas9 multiplex editing capability with the endogenous tRNA-processing system. Proceedings of the National Academy of Sciences 112: 3570-3575

Xu X, Chemparathy A, Zeng L, Kempton HR, Shang S, Nakamura M, Qi LS (2021) Engineered miniature CRISPR-Cas system for mammalian genome regulation and editing. Molecular cell 81: 4333-4345. e4334
